# Complete chloroplast genomes of 11 *Sabia* samples: Genomic features, comparative analysis, and phylogenetic relationship

**DOI:** 10.3389/fpls.2022.1052920

**Published:** 2022-12-16

**Authors:** Qiyu Chen, Chunling Chen, Bo Wang, Zehuan Wang, Wenfen Xu, Yuan Huang, Qingwen Sun

**Affiliations:** College of Pharmacy, Guizhou University of Traditional Chinese Medicine, Guiyang, China

**Keywords:** *Sabia*, chloroplast genome, comparative genomics, phylogenomics, divergence time

## Abstract

The genus *Sabia* is a woody climber belonging to the family Sabiaceae, order Proteales. Several species of this genus have been utilized as medicines for treating diseases, such as rheumatic arthritis, traumatism, hepatitis, *etc*. However, the lack of molecular data has prevented the accurate identification and refinement of taxonomic relationships in this genus. In this study, chloroplast genomes of 11 samples of the genus *Sabia* were assembled and analyzed. These chloroplast genomes showed a typical quadripartite structure and ranged in length from 160,956 to 162,209 bp. The structure of the genomes was found to be relatively conserved, with 130 genes annotated, including 85 coding genes, 37 tRNA genes, and eight rRNA genes. A total of 78–98 simple sequence repeats and 52–61 interspersed repeats were detected. Sequence alignment revealed 11 highly variable loci in chloroplast genomes. Among these loci, *ndhF*-*ndhD* achieved a remarkably higher resolution than the other regions. In addition, phylogenetic analysis indicated that Sect. *Pachydiscus* and Sect. *Sabia* of *Sabia* did not form two separate monophyletic groups. The divergence time calculated based on the Reltime method indicated that the evolutionary branches of *Sabia* and *Meliosma* started to form approximately 85.95 million years ago (Mya), and the species within *Sabia* began to diverge approximately 7.65 Mya. In conclusion, our study provides a basis for comprehensively exploring the phylogenetic relationships of *Sabia*. It also provides a methodological basis and data support for establishing a standardized and scientific identification system for this genus.

## Introduction

*Sabia* is a genus that belongs to the family Sabiaceae, order Proteales, and is a relatively basal group of Eudicots. This genus consists of woody climbers and scandent shrubs mainly distributed in the tropics of southern and southeast Asia along with some species spreading to the temperate zone (Flora of China Editorial Committee, 1985; The Angiosperm Phylogeny Group, 2016). While interacting with the natural environment, humans living in southern China discovered special uses of this genus. The roots, stems, and leaves of some species within the genus *Sabia* are used as traditional medicines owing to their clear curative effects on rheumatic arthritis, trauma, hepatitis, and other diseases. Recent studies have revealed that the main constituents of *Sabia* are terpenoids, alkaloids, flavonoids, *etc*., which exert a range of beneficial effects, including hepatoprotective, anti-inflammatory, antiviral, and other pharmacological effects (Wen et al., 2016; Chen et al., 2022b). Moreover, due to its excellent antioxidant activity, *Sabia parviflora* Wall. is consumed as green tea (Chen et al., 2020). Thus, it is clearly evident that this medicinal and edible genus has a substantial economic value.

The classification of the family Sabiaceae within angiosperms has long been controversial. Based on morphological classification, the family Sabiaceae has been placed in different systematic positions. Since its description, the family has been associated with Anacardiaceae and Sapindaceae based in part on the characteristics of small flowers and drupaceous fruits, *etc*. (Zuniga, 2015). Cronquist’s System of Classification placed Sabiaceae within the order Ranunculales on the basis of its pollen morphology and embryology (Cronquist, 1988). In APG, APG II, and APG III, the family Sabiaceae was not placed in any order (The Angiosperm Phylogeny Group, 1998; The Angiosperm Phylogeny Group, 2003; The Angiosperm Phylogeny Group, 2009). Some studies placed Sabiaceae as a sister taxon to the order Proteales but usually with low to moderate support (Moore et al., 2010; Ruhfel et al., 2014). Until 2016, Sun et al. (2016) conducted a phylogenetic analysis using protein-coding regions of the chloroplast genomes. The result showed that species of the family Sabiaceae (*Meliosma* aff. *cuneifolia* Franch. and *Sabia yunnanensis* Franch.) were clustered with species of the order Proteales, providing a strong support for a clade containing Sabiaceae and Proteales. Finally, in APG IV, Sabiaceae was moved into Proteales (The Angiosperm Phylogeny Group, 2016). Although the classification problem of family Sabiaceae has been resolved, phylogenetic relationships within the genus *Sabia* still remain controversial.

The first species of the genus *Sabia* was introduced by Colebrooke in 1819, when the genus contained only *Sabia lanceolata* Colebr. In the following century, species with morphological characteristics similar to those of *S. lanceolata* were discovered. In 1943, L. Chen systematically revised the genus *Sabia* to contain 53 species (Chen, 1943). Based on the characteristics of disc, Chen divided the genus *Sabia* into Sect. *Pachydiscus* and Sect. *Odontodiscus*. In 1980, V. D. Water’s revision considered that “a distinct subdivision of the genus into well-delimited sections, reflecting more or less natural affinities, is not well possible.” Therefore, the species of Sect. *Pachydiscus* established by Chen were reduced to a single species, *Sabia campanulata* Wall. ex Roxb., and the genus was revised to contain 19 species (Water, 1980). In the subsequent compilation of Flora Reipublicae Popularis Sinicae, Water’s revision of Sect. *Pachydiscus* was considered inappropriate, and Chen’s classification method based on disc characteristics was retained, with two sections renamed as Sect. *Pachydiscus* and Sect. *Sabia* (Liu and Wu, 1982; Flora of China Editorial Committee, 1985). In the 2007 revision of Flora of China, the number of *Sabia* species was recorded as approximately 30 worldwide, with 17 in China (Guo and Anthony, 2007).

Species within the genus are undoubtedly similar in appearance. Many of the species in the genus *Sabia* are difficult to identify accurately without the reproductive organs. However, the small-sized flowers, short flowering period, and low fruiting rate make *Sabia* difficult to identify in practice, which not only hampers the refinement of taxonomic relationships of the genus but also affects the exploitation of this medicinal and potentially edible genus. Thus, there is a need to conduct accurate taxonomy and identification studies and explore the applicability of different methods for identifying the species of this genus. Previous identification studies of *Sabia* mainly focused on microscopic or chemical features (Wen et al., 2016; Chen et al., 2022b). Identification studies based on molecular markers, such as *trnH*-*psbA*, *psbK*-*psbI*, *matK*, *rbcL*, and ITS2, were conducted (Sui et al., 2011; Yan et al., 2020a; Yan et al., 2020b), but these short DNA fragments lacked resolution, presenting an obstacle to complete resolution of the taxonomy and identification of this genus.

Chloroplasts are important organelles in plants that sustain life on earth by converting solar energy to carbohydrates through photosynthesis and oxygen release. They also play significant roles in biosynthesis, carbon fixation, and stress response (Neuhaus and Emes, 2000; Daniell et al., 2016). Chloroplasts are semiautonomous organelles with genome containing its own genetic system (Wolfe et al., 1987), which is the second largest genome in plant cells. Given its uniparental inheritance, moderate mutation rate, and relatively convenient sequencing, the chloroplast genome is often accepted as a more effective resource than the nuclear and mitochondrial genomes for exploring the origin and evolution of plants, understanding the phylogenetic relationships of different taxonomic categories, and identifying species (Daniell et al., 2016; Dong et al., 2021; Chen et al., 2022a). Therefore, analyzing the chloroplast genome may be an effective approach to solve the problem of the taxonomic identification of plants within the genus *Sabia*.

To date, only two complete chloroplast genomes of the genus *Sabia* have been reported (Sun et al., 2016; Chen et al., 2021). This lack of gene data limits the ability to further explore this genus. In addition, the specific structural characteristics of *Sabia* chloroplast genomes and the evolutionary relationships among species within this genus remain unclear. In this study, we sequenced nine samples from eight species collected during field surveys in recent years. We also sequenced two samples found during surveys that were difficult to identify. Combined with the information published in the National Center for Biotechnology Information database, the chloroplast genomes of the genus *Sabia* were analyzed. In this study, we aimed to (1) characterize the basic structure of the chloroplast genomes of *Sabia*, (2) analyze the diversity of chloroplast genomes among species and identify hotspots with higher nucleotide diversity across species; and (3) preliminarily explore the phylogenetic relationships and divergence time of the genus *Sabia* based on chloroplast genomes. In this way, we aimed to provide a basis for further understanding of the evolutionary process and phylogenetic relationships of the genus *Sabia* and obtain data that can act as a foundation for further molecular marker development, molecular breeding, and other studies of this genus.

## Materials and methods

### Plant materials, DNA extraction, and sequencing

In this study, 11 samples of the genus *Sabia*, including eight species, two suspicious species, and one duplicate sample, were collected from different places in southern China (**Table 1**, **Supplementary Figure S1**). Fresh and healthy leaves of these 11 samples were collected and stored at −80℃. The specimens were deposited in the herbarium of Guizhou University of Traditional Chinese Medicine (GZUTCM). Total DNA was extracted from each sample using E.Z.N.A^®^ Plant DNA Kit (OMEGA Bio-tek, USA) according to the manufacturer’s instructions. Agarose gel (0.8%) and Nanodrop 2000 (Thermo Fisher Scientific, USA) were used to assess the quality and quantity of DNA. High-quality DNA was used to generate shotgun libraries with an average insert size of 350 bp. Sequencing was conducted on the Illumina NovaSeq 6000 System (Illumina, USA) to generate paired-end 2 × 150-bp reads. Approximately 4.67–8.62 Gb of raw data was obtained (**Table 2**).

**Table 1:** Information on the samples of genus *Sabia*.

Number	Species	Code	Location	GenBank accession number
1	*Sabia campanulata* subsp. *ritchieae* (Rehder & E.H. Wilson) Y.F. Wu	–	Guiyang, Guizhou, China	OP310790
2	*Sabia dielsii* H. Lév.	–	Longli County, Guizhou, China	OP310791
3	*Sabia fasciculata* Lecomte ex L. Chen	–	Dushan County, Guizhou, China	OP310792
4	*Sabia japonica* Maxim.	–	Xinyang city, Henan, China	OP310793
5	*Sabia limoniacea* Wall. ex Hook. f. & Thomson	–	Zhangzhou, Fujian, China	OP310794
6	*Sabia parviflora* Wall.	XH-1	Wangmo, Guizhou, China	OP310795
7	*Sabia schumanniana* Diels	–	Pu ‘an, Guizhou, China	OP310796
8	*Sabia swinhoei* Hemsl.	JY-1	Longli, Guizhou, China	OP310797
9	*Sabia swinhoei* Hemsl.	JY-2	Zheng ‘an, Guizhou, China	OP310798
10	*Sabia* sp.	CY-1	Changshun, Guizhou, China	OP310799
11	*Sabia* sp.	CY-2	Malipo, Yunnan, China	OP310800

**Table 2:** Features of 11 *Sabia* chloroplast genomes.

	*S. campanulata* subsp. *ritchieae*	*S. dielsii*	*S. fasciculata*	*S.japonica*	*S. limoniacea*	*S. parviflora*(XH-1)	*S. schumanniana*	*S. swinhoei* (JY-1)	*S. swinhoei* (JY-2)	*S.* sp. (CY-1)	*S.* sp. (CY-2)
Raw reads (bp)	4,673,473,500	5,050,143,900	5,075,034,300	5,083,104,300	4,995,543,300	8,096,884,200	5,163,537,900	5,066,805,300	8,623,398,300	5,043,597,300	7,558,473,900
Depth (X)	1,355	1,105	1,365	1,753	1,174	2,955	2,478	1,017	2,097	1,046	4,477
Genome size (bp)	162,064	160,970	161,602	162,209	161,621	162,009	162,030	161,592	161,592	160,956	161,583
GC (%)	38.61	38.73	38.61	38.56	38.60	38.64	38.61	38.59	38.59	38.73	38.61
LSC size (bp)	89,980	88,990	89,906	90,048	89,990	89,971	89,974	89,862	89,862	88,986	89,931
GC in LSC (%)	37.04	37.19	37.10	36.99	37.10	37.07	37.04	37.07	37.07	37.19	37.11
SSC size (bp)	18,904	18,772	18,976	18,979	18,909	18,888	18,920	19,008	19,008	18,762	18,930
GC in SSC (%)	33.31	33.46	33.29	33.21	33.24	33.38	33.31	33.33	33.33	33.47	33.27
IR size (bp)	26,590	26,604	26,360	26,591	26,361	26,575	26,568	26,361	26,361	26,604	26,361
GC in IR (%)	43.16	43.17	43.09	43.12	43.09	43.16	43.15	43.08	43.08	43.17	43.09
1st position GC (%)	46.21	46.27	46.15	46.20	46.17	46.24	46.21	46.15	46.15	46.28	46.18
2^nd^ position GC (%)	38.77	38.79	38.80	38.74	38.78	38.78	38.78	38.80	38.80	38.79	38.78
3^rd^ position GC (%)	31.67	31.78	31.73	31.71	31.75	31.70	31.70	31.72	31.72	31.77	31.73
Number of CDS	85	85	85	85	85	85	85	85	85	85	85
Length of CDS	79,203	79,197	79,221	79,209	79,215	79,242	79,203	79,263	79,263	79,197	79,215
Number of tRNA	37	37	37	37	37	37	37	37	37	37	37
Length of tRNA	2,789	2,789	2,789	2,789	2,789	2,789	2,789	2,789	2,789	2,789	2,789
Number of rRNA	8	8	8	8	8	8	8	8	8	8	8
Length of rRNA	9,050	9,050	9,050	9,050	9,050	9,050	9,050	9,050	9,050	9,050	9,050

### Chloroplast genome assembly and annotation

Trimmomatic v0.39 (Bolger et al., 2014) was used to remove adapter-containing sequences and low-quality reads. NOVOPlasty v4.2 (Dierckxsens et al., 2017) was used to assemble chloroplast genome sequences with default parameters, except for the seed input. Approximately 2 million reads were randomly selected and mapped to the reference sequence of *Sabia yunnanensis* (NC_029431) using BWA v0.7.15 (Li and Durbin, 2009) (mem algorithm, default parameters), and a perfect-matched read to the *psbA* gene was selected as the seed input. The sequences were initially annotated using CpGAVAS 2 (Shi et al., 2019) and GeSeq (Tillich et al., 2017) and corrected manually. tRNAs were annotated using tRNAscan-SE (Lowe and Chan, 2016). Chloroplot (Zheng et al., 2020) was used to generate the circular chloroplast genome map. The annotated genome sequences were deposited in GenBank with accession numbers OP310790–OP310800 (**Table 1**).

### IR boundary and repeat sequence

A comparative analysis of inverted repeat (IR) boundaries was performed by combining data from 11 chloroplast genomes sequenced in this study and three Sabiaceae species, including *Sabia yunnanensis* (NC_029431.1), *Sabia parviflora* (NC_059863.1, coded XH-2), and *Meliosma* aff. *cuneifolia* (Sabiaceae, NC_029430.1). IRscope (Amiryousefi et al., 2018) was used to perform visual analysis. Simple sequence repeats (SSRs) and interspersed repeats of the chloroplast genomes of *Sabia* were detected from 13 samples, including samples sequenced in this study and downloaded sequences of *S. yunnanensis* (NC_029431.1) and *S. parviflora* (NC_059863.1). MISA (Beier et al., 2017) was used to detect SSRs with minimal repeat units set as 10 for mononucleotide SSRs, five for dinucleotide SSRs, four for trinucleotide SSRs, and three for tetranucleotide, pentanucleotide, and hexanucleotide SSRs. The length between two SSRs was set as 0. Interspersed repeats, including forward repeats, palindromic repeats, reverse repeats, and complementary repeats, were detected using REPuter (Kurtz et al., 2001) with a minimal repeat size set as 30 bp, along with a hamming distance set as 3.

### Chloroplast genome comparison

Combined with *S. yunnanensis* and *S. parviflora* (XH-2), the highly variable regions of 13 chloroplast genomes of *Sabia* were analyzed. mVISTA (Frazer et al., 2004) was used to perform a visual analysis with LAGAN model, setting *S. yunnanensis* as the reference sequence. Moreover, sliding window analysis was conducted to determine the nucleotide variability (Pi) of the complete chloroplast genome using DnaSP v6 (Rozas et al., 2017) after sequence alignment with MAFFT v7.471 (Katoh and Standley, 2013). The sliding window length was set as 600 bp, with a step size of 200 bp. In addition, DnaSP v6 was adopted to calculate insertions and deletions (InDels) and Pi for highly variable regions. Variable sites and parsimony information sites were analyzed using MEGA 11 (Tamura et al., 2021). The four universal chloroplast DNA barcodes *matK*, *psbK-psbI*, *rbcL*, and *trnH-psbA* were used in this analysis.

### Phylogenetic analysis

Phylogenetic analysis was performed using single-copy regions and one inverted repeat region of 13 chloroplast genomes of *Sabia*. *Meliosma* aff. *cuneifolia* (NC_029430.1) was set as the outgroup. After sequence alignment using MAFFT, the index of substitution saturation (*I*_ss_) was evaluated using DAMBE v5.3.19 (Xia, 2013): *I*_ss_ (0.1228) < *I*_ss.c_ (0.8410), *P* = 0.0000. The substitution of the 13 chloroplast genomes was not saturated. A maximum likelihood (ML) phylogenetic tree was constructed using IQ-TREE v1.6.12 (Nguyen et al., 2015) under the TVM+F+R2 best-fit model selected by Modelfinder of IQ-TREE, and 1,000 bootstrap replications were used to estimate the statistical reliability of each branch. A maximum parsimony (MP) phylogenetic tree was constructed using MEGA 11 with the Tree-Bisection-Reconnection search method. Bootstrap values were calculated with 1,000 replications. A Bayesian inference (BI) phylogenetic tree was constructed using MrBayes v3.2.7 (Huelsenbeck and Ronquist, 2001) under the GTR+I+G nucleotide substitution model selected by MrModeltest v2.3. Four Markov chains were run for 2 million generations, with trees sampled every 1,000 generations. After 25% of aging samples were discarded, a consensus tree with posterior probabilities was constructed using the remaining samples.

The genetic distances between *Sabia* species were also calculated. After sequence alignment of the 13 chloroplast genome sequences using MAFFT, the genetic distances between species were calculated using MEGA 11 with the Kimura two-parameter model and 1,000 bootstrap replications.

### Divergence time estimation

According to APG IV (The Angiosperm Phylogeny Group, 2016), the family Sabiaceae belongs to the order Proteales, which has a close genetic relationship with Ranunculales and other Eudicots groups. On this basis, 13 chloroplast genomes of *Sabia*, five species of *Meliosma*, and 12 other species in the order Proteales were selected for the estimation of divergence time, setting *Semiaquilegia guangxiensis* Yan Liu & Y. S. Huang (family Ranunculaceae, order Ranunculales) and *Epimedium ecalcaratum* G.Y. Zhong (family Berberidaceae, order Ranunculales) as outgroups (**Supplementary Table S1**). After MAFFT alignment and substitution saturation testing [*I_ss_* (0.7066) < *I_ss.c_* (0.8135), *P* = 0.0000] of 32 chloroplast genome sequences, IQ-TREE was used to construct an ML phylogenetic tree based on the TVM+F+R4 nucleotide substitution model. The divergence time was estimated using the RelTime (Tamura et al., 2012) method in MEGA 11 with GTR model. The divergence times between *Nelumbo nucifera* Gaertn. and *Nelumbo lutea* Willd.(1.5–11.8 Mya), *Macadamia integrifolia* Maiden & Betche and *Platanus occidentalis* L. (81.5–114 Mya), and *Nelumbo lutea* and *Platanus occidentalis* (105–119.6 Mya) obtained in Timetree (Kumar et al., 2017) (www.timetree.org) were used as calibration constraints for estimation (**Supplementary Figure S2**).

## Results

### Chloroplast genome features

Eleven chloroplast genomes of the genus *Sabia* were assembled, which ranged in length from 160,956 to 162,209 bp. These chloroplast genomes presented a typical quadripartite structure with double-stranded DNA, including a large single-copy (LSC) region ranging from 88,986 to 90,048 bp, a small single-copy (SSC) region ranging from 18,762 to 19,008 bp, and two inverted repeat (IRa and IRb) regions ranging from 26,360 to 26,604 bp. The total GC contents of the 11 samples were similar among species, within the range of 38.56%–38.73%. However, the GC content varied among different regions of the genome, with 36.99%–37.19% in the LSC region, 33.21%–33.47% in the SSC region, and 43.08%–43.17% in the IR region, with all cases showing the following order: SSC < LSC < IR (**Table 2**).

**Figure 1:**
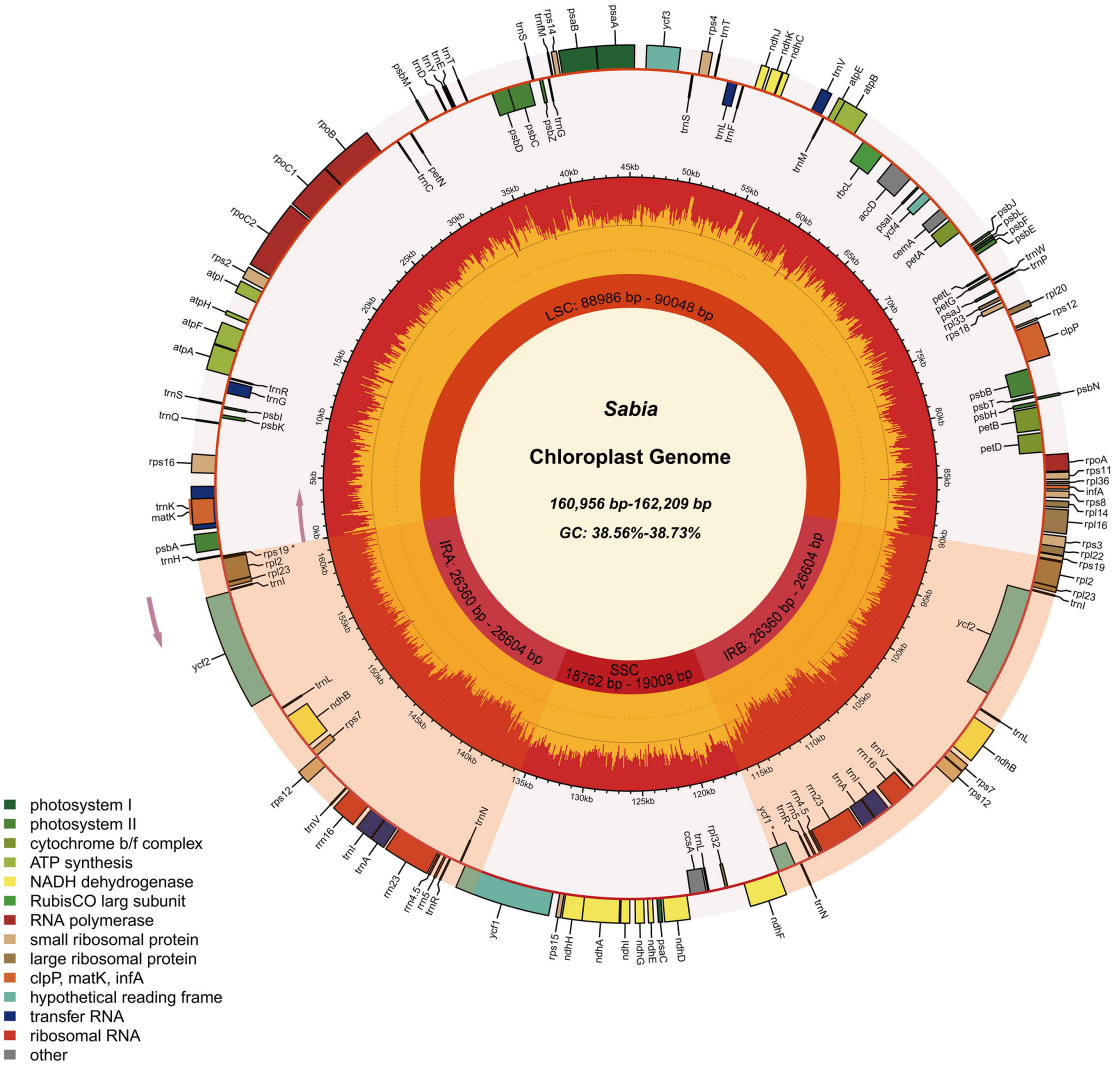
Chloroplast genome map of 11 *Sabia* samples. In the first inner circle, the lengths of the corresponding large single-copy region, small single-copy region, and two inverted repeat regions of the chloroplast genomes of *Sabia* are given. In the second circle, the orange area denotes the GC content of each region, and the yellow area corresponds to the AT content. The distribution of genes is shown in the outermost circle, with pseudogenes marked with an asterisk. Genes are colored according to their function. The direction of transcription for the inner circle genes is clockwise, while that for the outer circle genes is anticlockwise.

A total of 130 genes, including 85 coding genes, 37 transport RNA (tRNA) genes, and eight ribosomal RNA (rRNA) genes, were annotated in each of the 11 chloroplast genomes. The order and orientation of these genes were the same in all 11 chloroplast genomes (**Figure 1**). Coding sequences (CDSs), tRNA, and rRNA genes were 79,197–79,263, 2,789, and 9,050 bp in length, accounting for 48.83%–49.20%, 1.72%–1.73%, and 5.58%–5.62% of the entire genomes, respectively. This indicates that approximately 43.44%–43.87% of the genomes comprised noncoding regions (**Table 2**).

Among the 130 genes annotated, six coding genes (*rps12*, *rps7*, *rpl23*, *rpl2*, *ndhB*, and *ycf2*), seven tRNA genes (*trnA*-*UGC*, *trnI*-*CAU*, *trnI*-*GAU*, *trnL*-*CAA*, *trnN*-*GUU*, *trnR*-*ACG*, and *trnV*-*GAC*), and all rRNA genes (*rrn16*, *rrn23*, *rrn4.5*, and *rrn5*) included two repeat units due to their presence in the IR region. A total of 19 genes, including 11 coding genes (*atpF*, *ndhA*, *ndhB*×2, *petB*, *petD*, *rpl16*, *rpl2*×2, *rpoC1*, and *rps16*) and eight tRNA genes (*trnA*-*UGC*×2, *trnG*-*UCC*, *trnI*-*GAU*×2, *trnK*-*UUU*, *trnL*-*UAA*, and *trnV*-*UAC*), contained one intron. A total of four genes contained two introns (*rps12*×2, *clpP*, and *ycf3*) (**Table 3**). In addition, two genes (*ycf1*, and *rps19*) were annotated as pseudogenes, and *rps12* was a trans-splicing gene with divided parts: the 5′ end located in the LSC region and the 3′ end in the IR region.

**Table 3:** Gene contents in the *Sabia* chloroplast genomes.

Category of genes	Group of genes	Name of genes	Number
Genes for the genetic system	Ribosomal RNAs	*rrn16* (×2), *rrn23* (×2), *rrn4.5* (×2), *rrn5* (×2)	8
	Transfer RNAs	*trnA*-*UGC* (×2)^a^, *trnC*-*GCA*, *trnD*-*GUC*, *trnE*-*UUC*, *trnF*-*GAA*, *trnfM*-*CAU*, *trnG*-*GCC*, *trnG*-*UCC* ^a^, *trnH*-*GUG*, *trnI*-*CAU* (×2), *trnI*-*GAU* (×2)^a^, *trnK*-*UUU* ^a^, *trnL*-*CAA* (×2), *trnL*-*UAA* ^a^, *trnL*-*UAG*, *trnM*-*CAU*, *trnN*-*GUU* (×2), *trnP*-*UGG*, *trnQ*-*UUG*, *trnR*-*ACG* (×2), *trnR*-*UCU*, *trnS*-*GCU*, *trnS*-*GGA*, *trnS*-*UGA*, *trnT*-*GGU*, *trnT*-*UGU*, *trnV*-*GAC* (×2), *trnV*-*UAC* ^a^, *trnW*-*CCA*, *trnY*-*GUA*	37
	Small subunit of ribosome	*rps11*, *rps12* (×2)^b^, *rps14*, *rps15*, *rps16* ^a^, *rps18*, *rps19*, *rps2*, *rps3*, *rps4*, *rps7* (×2), *rps8*	14
	Large subunit of ribosome	*rpl14*, *rpl16* ^a^, *rpl20*, *rpl22*, *rpl23* (×2), *rpl2* (×2)^a^, *rpl32*, *rpl33*, *rpl36*	11
	DNA-dependent RNA polymerase	*rpoA*, *rpoB*, *rpoC1* ^a^, *rpoC2*	4
Genes for the photosynthetic system	Subunits of NADH dehydrogenase	*ndhA* ^a^, *ndhB* (×2)^a^, *ndhC*, *ndhD*, *ndhE*, *ndhF*, *ndhG*, *ndhH*, *ndhI*, *ndhJ*, *ndhK*	12
	Subunits of photosystem I	*psaA*, *psaB*, *psaC*, *psaI*, *psaJ*	5
	Subunits of photosystem II	*psbA*, *psbB*, *psbC*, *psbD*, *psbE*, *psbF*, *psbH*, *psbI*, *psbJ*, *psbK*, *psbL*, *psbM*, *psbN*, *psbT*, *psbZ*	15
	Assembly protein of photosystem I	*ycf3* ^b^, *ycf4*	2
	Subunits of cytochrome b/f complex	*petA*, *petB* ^a^, *petD* ^a^, *petG*, *petL*, *petN*	6
	Subunits of ATP synthase	*atpA*, *atpB*, *atpE*, *atpF* ^a^, *atpH*, *atpI*	6
	Large subunit of rubisco	*rbcL*	1
Genes for the biosynthesis	Maturase	*matK*	1
	Protease	*clpP* ^b^	1
	Envelope membrane protein	*cemA*	1
	Subunit of acetyl-CoA-carboxylase	*accD*	1
	C-type cytochrome synthesis gene	*ccsA*	1
	Translational initiation factor	*infA*	1
Genes of unknown function	Open reading frames	*ycf1*, *ycf2* (×2)	3

(×2) indicates that a gene contains two repeating units.^a^Gene containing one intron.

^b^Gene containing two introns.

The 85 coding genes of the 11 chloroplast genomes consisted of 26,399–26,421 codons encoding 20 amino acids and stop codons. Among these, codons for leucine (Leu) were the most abundant (10.25%–10.27%), and those for cysteine (Cys) were the least (1.17%–1.19%). The number of codons was relatively conserved with no significant differences among the species (**Supplementary Table S2**). Furthermore, in CDSs, the GC content of the first, second, and third codon positions was 46.15%–46.28%, 38.74%–38.80%, and 31.67%–31.78%, respectively, for all cases with the GC content in the following order: third position < second position < first position (**Table 2**).

### Contraction and expansion of IR boundaries

**Figure 2:**
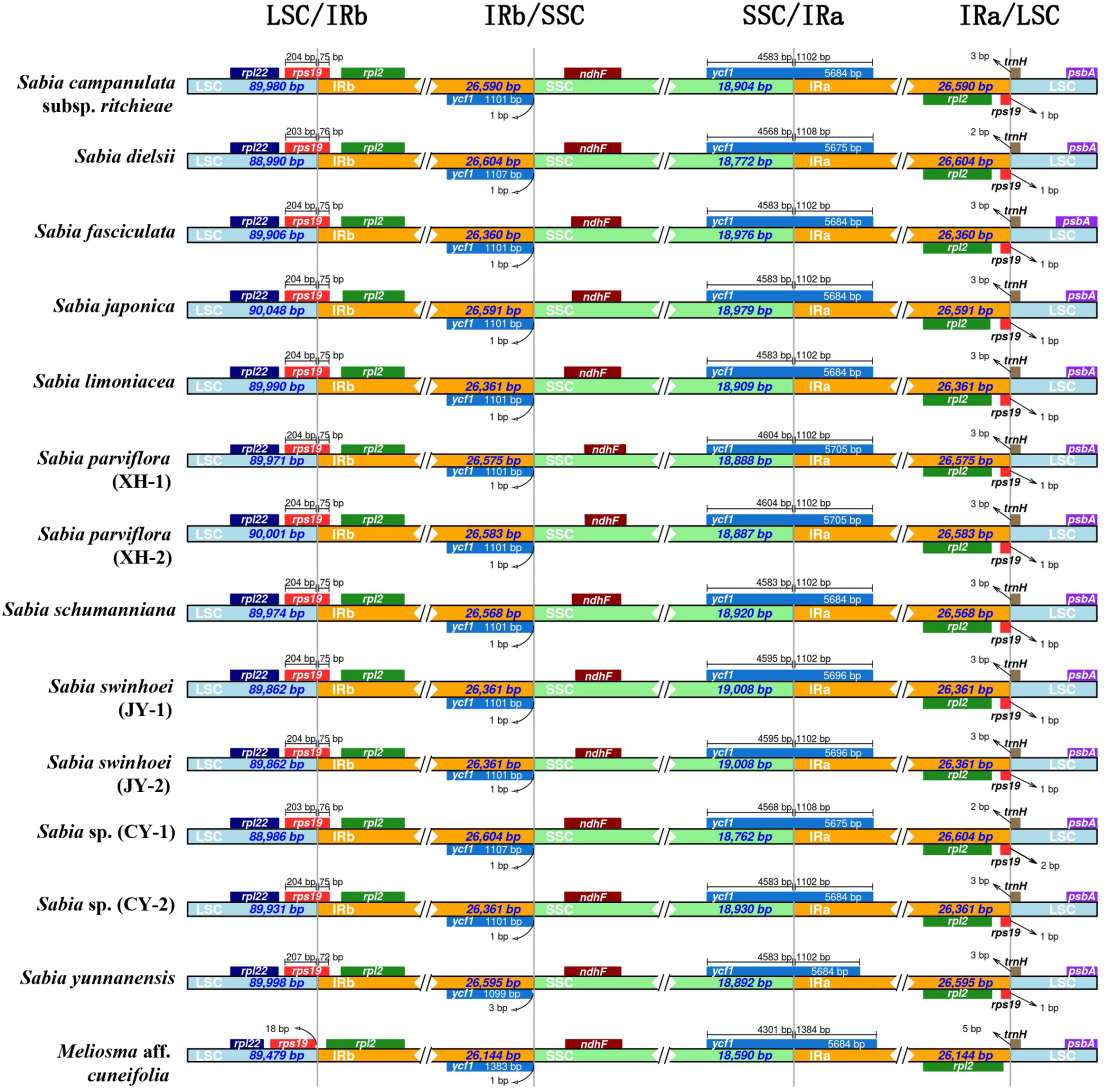
Comparison of small single-copy region (SSC), large single-copy (LSC), and inverted repeat regions (IRa and IRb) boundaries in 14 chloroplast genomes of Sabiaceae. LSC/IRb, IRb/SSC, SSC/IRa, and IRa/LSC denote the junction sites between each corresponding two regions of the genomes.

The length of the IR regions in the 13 chloroplast genomes of *Sabia* ranged from 26,360 to 26,604 bp, and the length of the IR region of the chloroplast genome of *M.* aff. *cuneifolia* was 26,144 bp. In the genus *Sabia*, the LSC/IRb boundaries were located in *rps19* genes, which had 72, 75, or 76 bp in the IRb regions. This led to the detection of incomplete *rps19* pseudogenes in the IRa regions. However, the IRb region did not expand into the *rps19* gene of *M.* aff. *cuneifolia* and was separated from the LSC/IRb boundary by 18 bp. In all tested samples, the SSC/IRa boundaries were in *ycf1*, which had 1,108 bp in the IRa regions for *S. dielsii* and *S.* sp. (CY-1), 1,384 bp for *M.* aff. *cuneifolia*, and 1,102 bp for the others. Similarly, incomplete *ycf1* pseudogenes were discovered in the IRb regions. The *ndhF* and *trnH* genes of 14 chloroplast genomes were located in the SSC and LSC regions, respectively, and did not enter the IR regions (**Figure 2**).

### Repeat sequence

**Figure 3:**
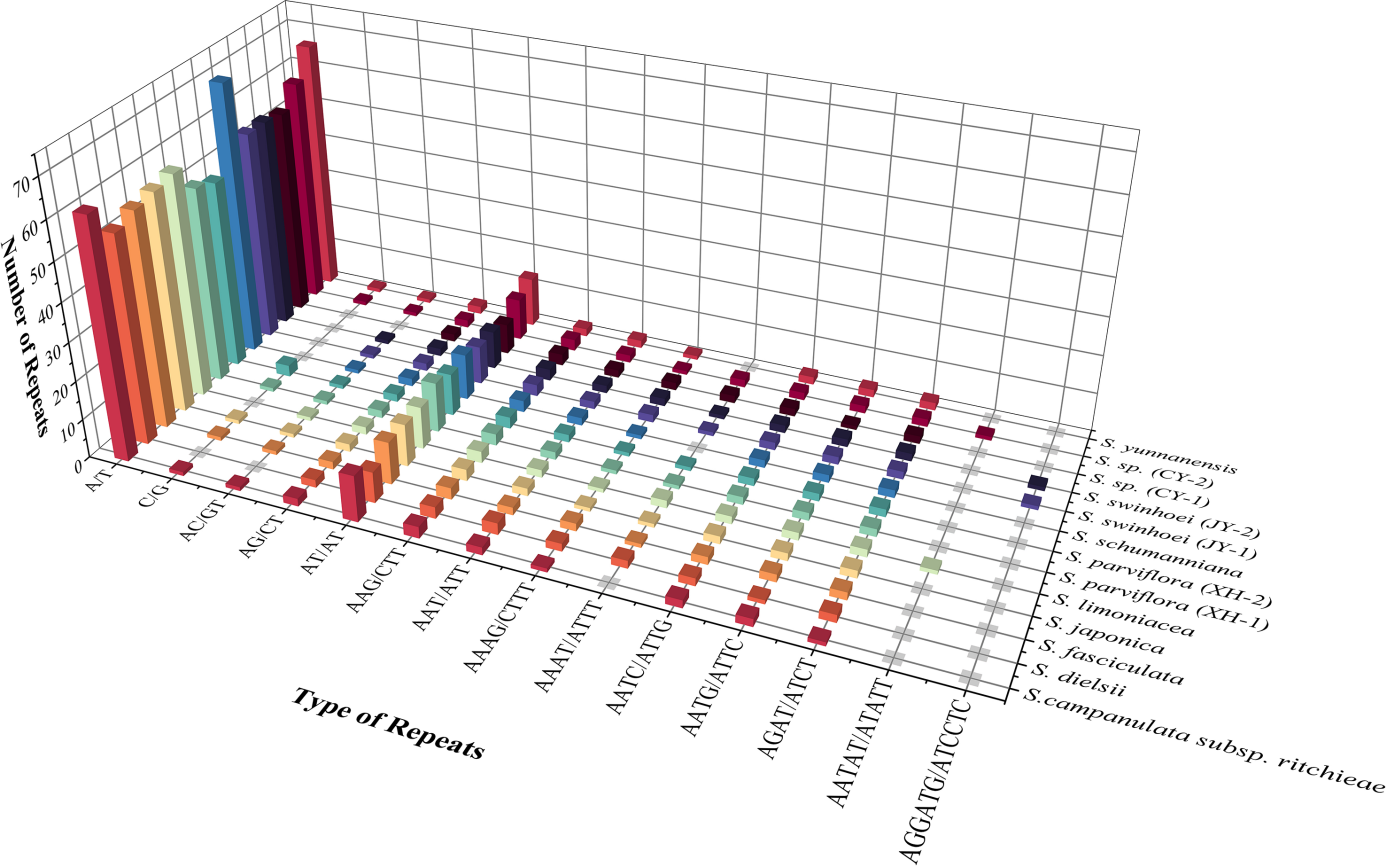
Type and number of simple sequence repeats in 13 *Sabia* chloroplast genomes.

SSRs are widespread in the chloroplast genome, with 1–6-bp repeat nucleotide units (Powell et al., 1995). A total of 78–98 SSRs were found in 13 chloroplast genomes of *Sabia*. All samples contained mononucleotide, dinucleotide, trinucleotide, and tetranucleotide SSRs, with only *S. limoniacea* and *S.* sp. (CY-2) containing pentanucleotide SSRs and two samples of *S. swinhoei* (JY-1 and JY-2) containing hexanucleotide SSRs. Among these SSRs, A/T mononucleotide SSRs were the most abundant, accounting for 62.03%–72.45% of the total SSRs, followed by AT/AT dinucleotide SSRs, which accounted for 10.13%–16.05% (**Figure 3**). This obvious AT bias is a common phenomenon in the chloroplast genome of higher plants (Lei et al., 2016; Zhou et al., 2018; Yang et al., 2019). Most of these SSRs were distributed in the intergenic spacers (IGSs) of LSC and SSC. They were also located in the coding regions of genes such as *rpoC2*, *ycf1*, *cemA*, *ndhE*, *ndhH*, and *rpl22* and intron regions of genes such as *ndhA*, *atpF*, *clpP*, *ycf3*, and *rpoC1* (**Table 4**).

**Table 4:** Number of simple sequence repeats in different regions of 13 *Sabia* chloroplast genomes.

	Intergenic spacers	Exon	Intron	Large single-copy	Small single-copy	Inverted repeat	Total
*S. campanulata* subsp*. ritchieae*	70	8	11	73	14	2	89
*S. dielsii*	59	11	9	67	12	0	79
*S. fasciculata*	61	11	13	70	13	2	85
*S. japonica*	57	11	17	67	14	4	85
*S. limoniacea*	69	9	9	74	11	2	87
*S. parviflora* (XH-1)	63	9	9	67	12	2	81
*S. parviflora* (XH-2)	63	9	7	66	11	2	79
*S. schumanniana*	75	8	15	81	15	2	98
*S. swinhoei* (JY-1)	59	10	14	68	13	2	83
*S. swinhoei* (JY-2)	60	9	14	68	13	2	83
*S.* sp. (CY-1)	58	11	9	66	12	0	78
*S.* sp. (CY-2)	71	8	10	75	12	2	89
*S. yunnanensis*	72	10	12	78	14	2	94
Proportion	67.06–79.78%	8.16–14.10%	8.86–20%	78.82–85.06%	12.64–16.47%	0–4.71%	–

**Figure 4:**
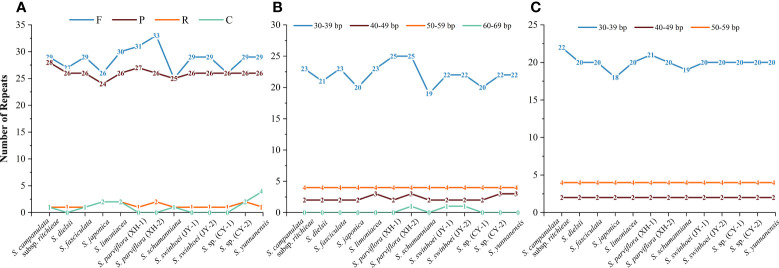
Interspersed repeats in 13 *Sabia* chloroplast genomes. **(A)** Number and type of interspersed repeats. F, forward repeats; P, palindrome repeats; R, reverse repeats; C, complementary repeats. **(B)** Frequency distribution of forward repeats. **(C)** Frequency distribution of palindrome repeats.

The number of interspersed repeats in the 13 chloroplast genomes of *Sabia* ranged from 52 to 61. *S. dielsii*, *S. parviflora* (XH-1 and XH-2), *S. swinhoei* (JY-1 and JY-2), and *S.* sp. (CY-1) contained only three types of interspersed repeat sequences: forward, palindrome, and reverse repeats, while the others contained four types: forward, palindrome, reverse, and complementary repeats (**Figure 4A**). Forward repeats (*n* = 25–33) and palindrome repeats (*n* = 24–28) were abundant, the lengths of which were particularly concentrated at 30–39 bp. Reverse repeats (*n* = 1 to 2) and complementary repeats (*n* = 0–4) were less in number, ranging in length from 30 to 39 bp (**Figures 4B, C**).

### Comparative chloroplast genome analysis

**Figure 5:**
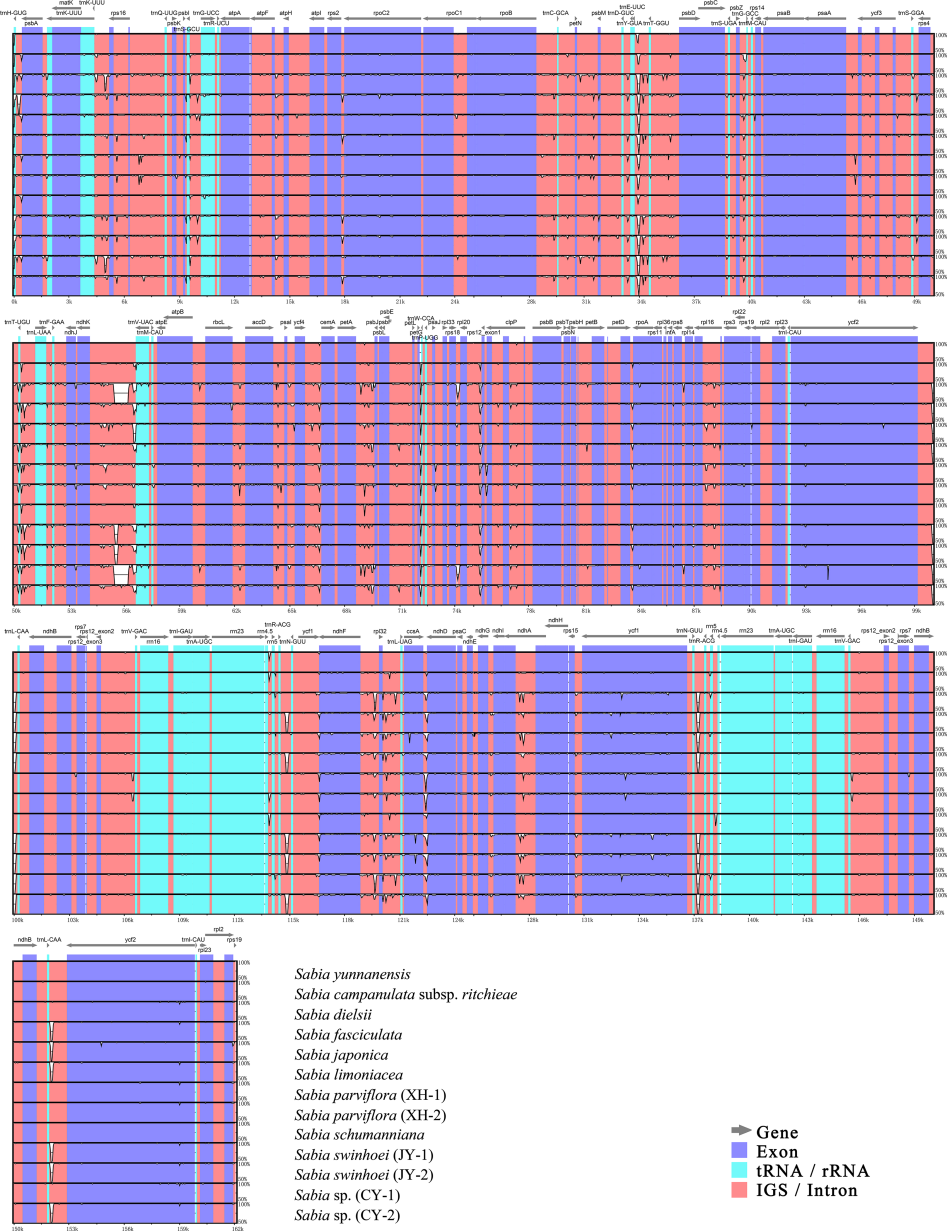
Complete chloroplast genome alignment of *Sabia* species with mVISTA. The vertical scale indicates the average percent identity, ranging from 50% to 100%. The horizontal scale indicates the coordinates within the chloroplast genome. Gray arrows above the alignment indicate genes with their orientation.

A plot enabling a comparative analysis of the 13 chloroplast genomes of *Sabia* was created using mVISTA, with the *S. yunnanensis* chloroplast genome as the reference (**Figure 5**). The chloroplast genomes were highly conserved among species, with significantly higher levels of variation in IGSs and intron regions than in exon regions. These regions with a higher variation were mainly located in *trnH*-*GUG*-*psbA*, *trnK*-*UUU*-*rps16*, *trnE-UUC*-*trnT-GGU*, *trnT-UGU*-*trnL-UAA*, *ndhK* (*ndhC*)-*trnV-UAC*, *petA*-*psbJ*, *petG*-*trnW-CCA*, *rpl20*-*rps12* (exon1), *ndhF*-*trnL*-*UAG*, *ccsA*-*ndhD*, *etc*.

**Figure 6:**
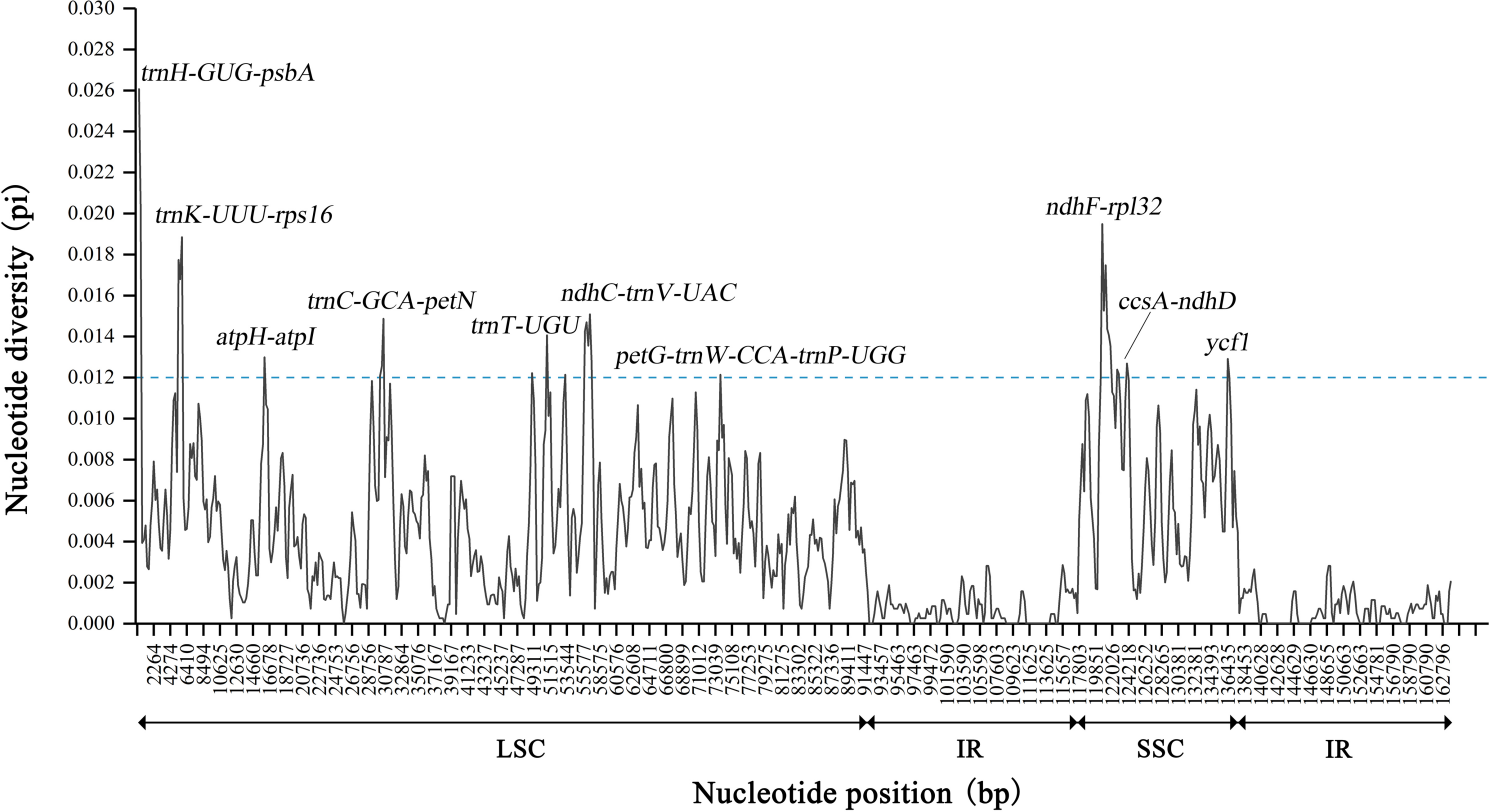
Nucleotide diversity of 13 *Sabia* chloroplast genomes. The X-axis represents the position of the chloroplast genome, and the Y-axis represents the nucleotide diversity of each window.

DnaSP was further used to detect highly variable regions in the 13 chloroplast genomes of *Sabia* (**Figure 6**). Similar to the results of the mVISTA analysis, the overall chloroplast genomes were conserved among species, with no large, highly variable regions. Two IR regions showed significant conservation compared with the LSC and SSC regions. The maximum Pi value was 0.02607, located near *trnH*-*GUG* to *psbA* gene in the LSC region. Moreover, in the LSC region, the regions near *trnS*-*GGA* and *trnT*-*UGU* as well as IGSs of *trnK*-*UUU*-*rps16*, *atpH*-*atpI*, *trnC*-*GCA*-*petN*, *trnF*-*GAA*-*ndhJ*, *ndhC*-*trnV*-*UAC*, and *petG*-*trnW-CCA*-*trnP*-*UGG* showed a higher nucleotide diversity among species, with Pi values greater than 0.012. In the SSC region, the largest diversity site was found between *ndhF* gene to *rpl32* gene, with a Pi value of 0.01949. Within the SSC region, from *ndhF* to *rpl32*, *trnL*-*UAG*, *ccsA*, and, finally, *ndhD* gene, there was a continuous highly variable region, except for exon regions. In addition, the rate of polymorphism of the *ycf1* gene was also high.

Based on nucleotide diversity analysis, 11 highly variable fragments were extracted and compared with the whole genome and four universal chloroplast DNA barcodes: *matK*, *psbK-psbI*, *rbcL*, and *trnH-psbA*. Undoubtedly, the chloroplast genome had the highest number of variable sites (*n* = 2,270) as well as parsimony information sites (*n* = 1,587) and InDels (*n* = 4,710) (**Table 5**). Among the highly variable fragments, *ndhF-ndhD* contained the highest number of variable sites (*n* = 186) and parsimony information sites (*n* = 137). *ndhC-trnV* contained the highest number of InDels (*n* = 982). In the four universal chloroplast DNA barcodes, the intergenic region of *trnH* gene to *psbA* gene showed better diversity than many fragments, but in general, most of the highly variable fragments were more variable than the four universal chloroplast DNA barcodes.

**Table 5:** Characteristics of the chloroplast genome and highly variable regions of *Sabia*.

	Regions	Aligned length (bp)	Variable sites (%)	Parsimony information sites (%)	InDels (%)	Nucleotide diversity (Pi)
	Genome	163,972	2,274 (1.39%)	1,587 (0.97%)	4,710 (2.87%)	0.00390
Highly variable regions	*trnH-psbA*	929	41 (4.41%)	34 (3.66%)	129 (13.89%)	0.01926
	*trnK-rps16*	1,087	43 (3.96%)	33 (3.04%)	85 (7.82%)	0.01343
	*atpH-atpI*	605	23 (3.80%)	20 (3.31%)	5 (0.83%)	0.01299
	*trnC-petN*	1,009	33 (3.27%)	25 (2.48%)	9 (0.89%)	0.01154
	*ycf3-trnS*	606	21 (3.47%)	19 (3.14%)	6 (0.99%)	0.01222
	*rps4-trnL*	758	35 (4.62%)	26 (3.43%)	158 (20.84%)	0.01406
	*trnF-ndhJ*	604	24 (3.97%)	18 (2.98%)	4 (0.66%)	0.01214
	*ndhC-trnV*	2,382	77 (3.23%)	60 (2.52%)	982 (41.23%)	0.01258
	*petG-trnP*	642	27 (4.21%)	22 (3.43%)	42 (6.54%)	0.01214
	*ndhF-ndhD*	3,999	186 (4.65%)	137 (3.43%)	399 (9.98%)	0.01282
	*ycf1*	812	27 (3.33%)	19 (2.34%)	12 (1.48%)	0.01061
Universal chloroplast DNA barcodes	*matK*	1,542	30 (1.95%)	21 (1.36%)	6 (0.39%)	0.00558
	*psbK-psbI*	423	5 (1.18%)	4 (0.95%)	16 (3.78%)	0.00416
	*rbcL*	1,428	20 (1.40%)	16 (1.12%)	0 (0.00%)	0.00523
	*trnH-psbA*	405	37 (9.14%)	32 (7.9%)	125 (30.86%)	0.05229

### Phylogenetic analysis

**Figure 7:**
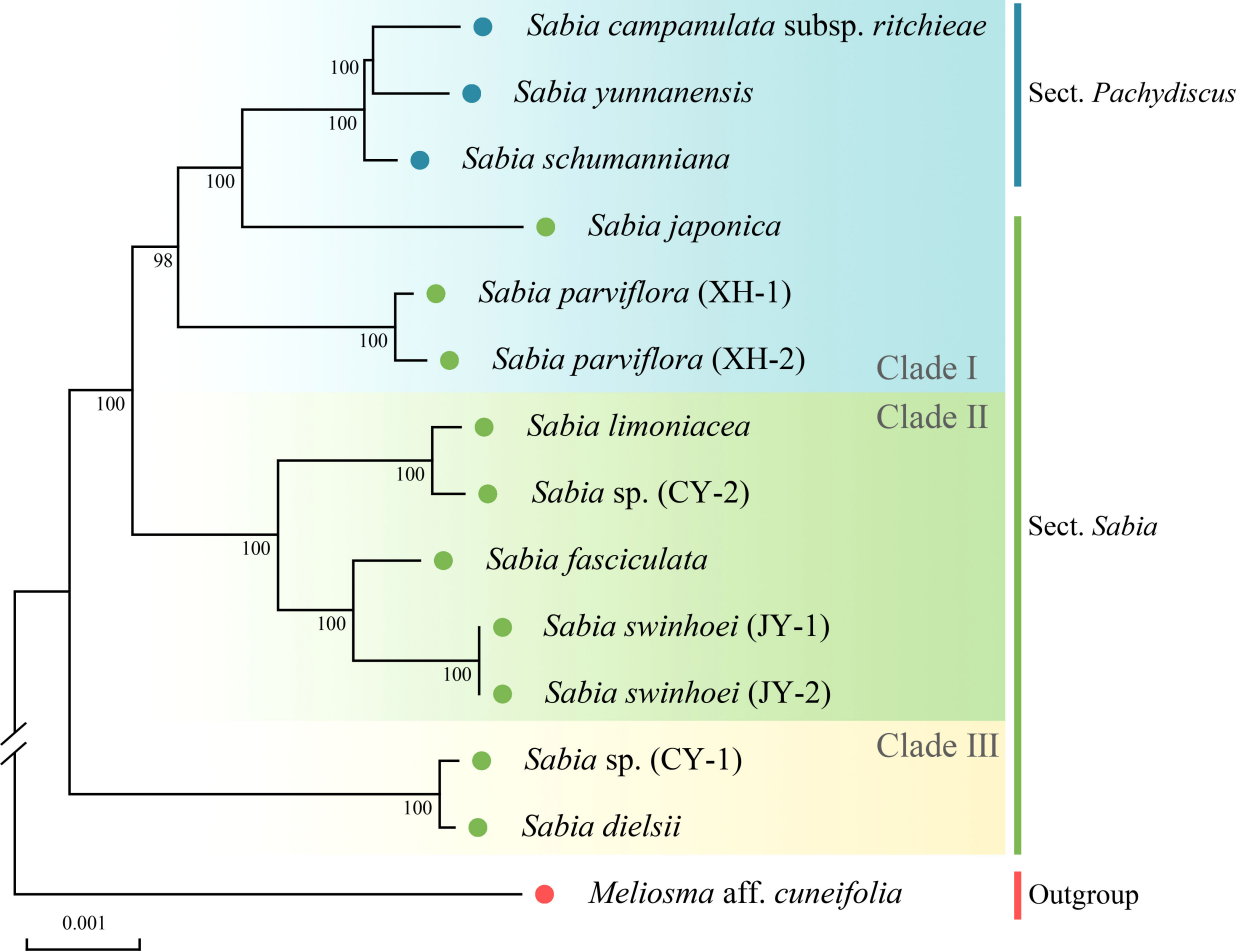
Phylogenetic tree obtained using the maximum likelihood method for *Sabia* species based on complete chloroplast genomes. Numbers above the branches indicate maximum likelihood bootstrap support values.

The phylogenetic trees constructed using the ML, MP, and BI methods shared the same topology, with each branch having high support values (**Figure 7**, **Supplementary Figure S3**). The phylogenetic tree of available *Sabia* species presented two main branches. One clade comprised *S.* sp. (CY-1) and *S. dielsii* (**Figure 7**, clade III), while the other clade was further divided into two subclades. One subclade contained *S. campanulata* subsp. *ritchieae*, *S. yunnanensis*, *S. schumanniana*, *S. japonica*, and *S. parviflora* (**Figure 7**, clade I). The other subclade contained *S. limoniacea*, *S.* sp. (CY-2), *S. fasciculata*, and *S. swinhoei* (**Figure 7**, clade II). Two samples each of *S. parviflora* and *S. swinhoei* were clustered into monophyletic groups.

Based on a morphological study (Flora of China Editorial Committee, 1985), species of the genus *Sabia* were divided into two sections: Sect. *Pachydiscus* and Sect. *Sabia*. *S. campanulata* subsp. *ritchieae*, *S. yunnanensis*, and *S. schumanniana* belonging to Sect. *Pachydiscus* were formed into a monophyletic group, which was consistent with the traditional morphological classification. However, the remaining samples belonging to Sect. *Sabia* did not form a monophyletic group.

**Figure 8:**
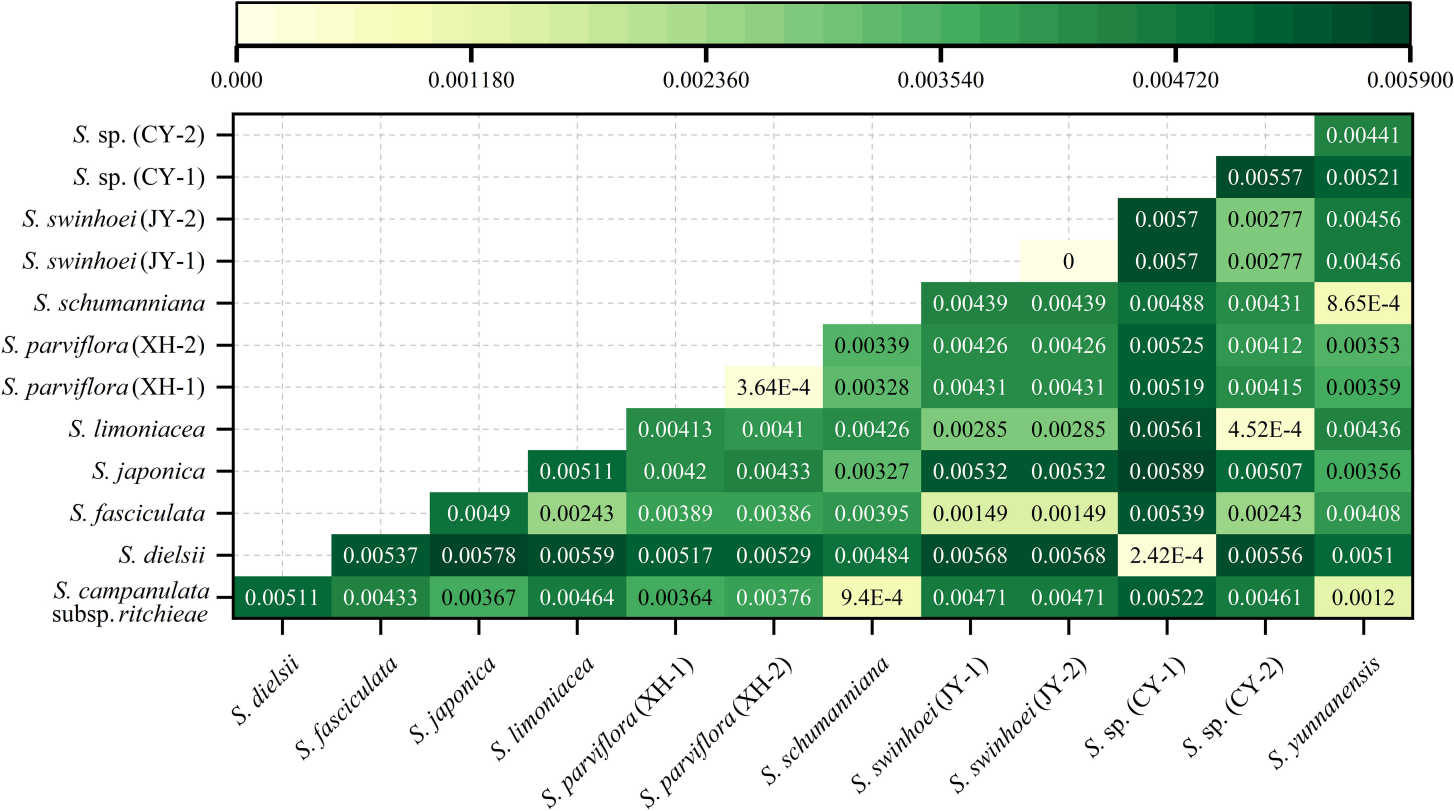
Genetic distances in chloroplast genomes of the genus *Sabia*. Distances from yellow to green and from light to dark indicate that the genetic relationships between species change from close to distant.

The genetic distances of the 13 chloroplast genomes ranged from 0.000000 (two samples of *S. swinhoei*) to 0.005888 [*S. japonica* and *S.* sp. (CY-1)] (**Figure 8**). These genetic distances between species were consistent with the results of phylogenetic trees. In addition, with the exceptions of *S. dielsii* and *S.* sp. (CY-1), the genetic distances between other samples were greater than those between two samples of *S. parviflora* (0.0003645).

### Divergence time estimation

**Figure 9:**
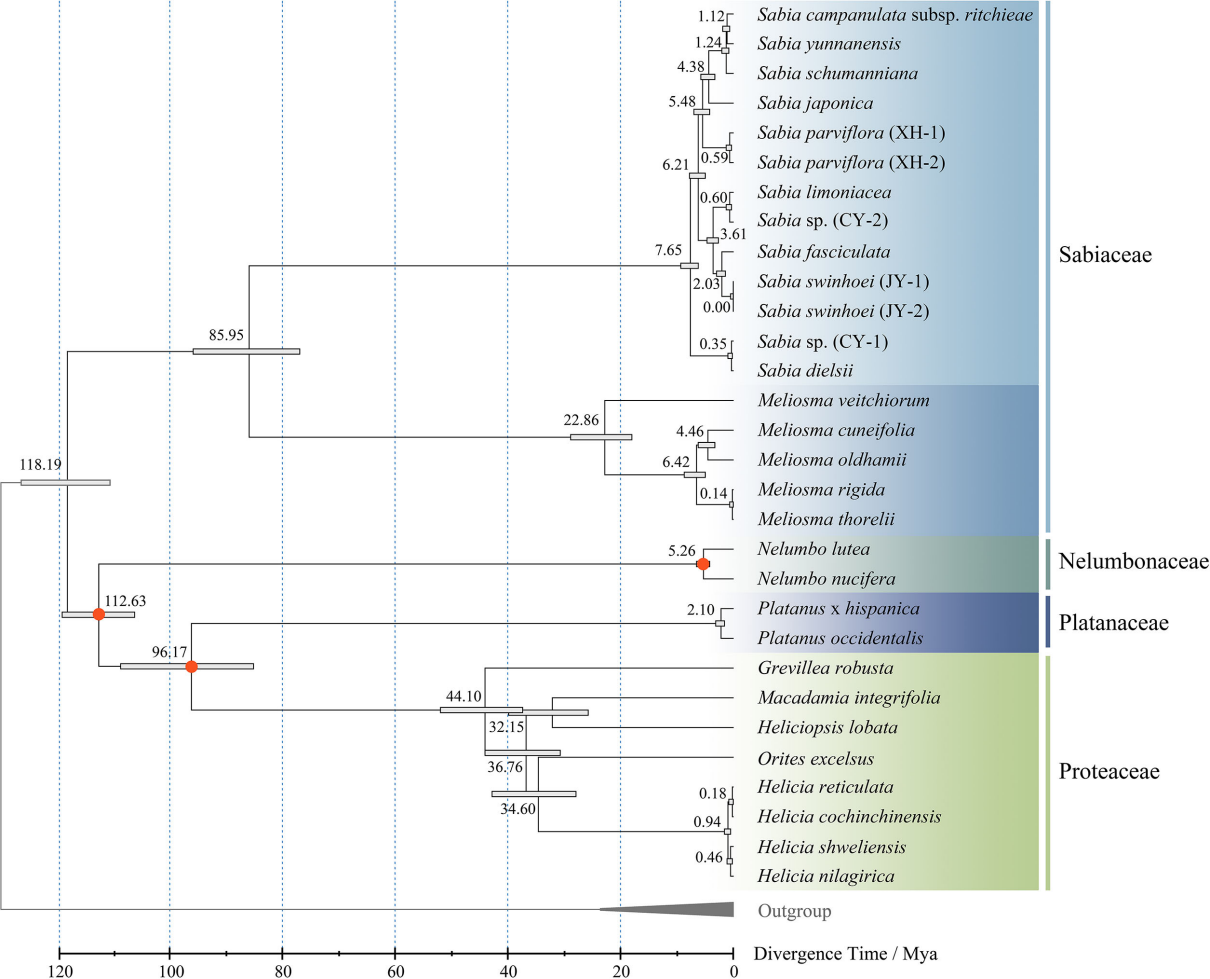
Divergence time estimation of genus *Sabia*. The numbers of the nodes represent the divergence time. Bars at the nodes represent 95% confidence intervals. Calibration points are marked with red.

Divergence time estimation was performed using Reltime method, with three pairs of estimated times as calibration constraints. The results showed that the divergence time between Sabiaceae and other families of the order Proteales was approximately 118.19 million years ago (Mya) during the Cretaceous. The two genera of family Sabiaceae diverged at approximately 85.95 Mya, which was similar to a previous estimate (Yang et al., 2018). The species within the genus *Sabia* began to diverge at approximately 7.65 Mya, suggesting that many species of this genus probably gradually emerged after the Cenozoic (**Figure 9**).

## Discussion

### Variations of complete chloroplast genomes in *Sabia*

In this study, the chloroplast genomes of 11 samples from genus *Sabia* were sequenced, assembled, and annotated using next-generation sequencing. The analyses showed that these chloroplast genome sequences were highly conserved in terms of genome structure, GC content, gene content, gene order, *etc*., without specific mutational structures within the genomes. Notably, the *ndhC* gene was not annotated in the chloroplast genome of *S. yunnanensis*. However, blast alignment identified a sequence matching to *ndhC* in *S. yunnanensis* chloroplast genome. In addition, phenomena that occur in the chloroplast genomes of *Sabia*, such as the uneven distribution of GC content across regions and trans-splicing of *rps12*, were ubiquitous in most plants (Wang et al., 2018; Thode and Lohmann, 2019; Lu et al., 2022), and no specific structural variation has been found in this genus.

The IR region is a commonly found region in most higher plants, and its contraction and expansion is a common evolutionary event that is considered to be one of the main reasons for the variation in size of the chloroplast genome (Wang et al., 2008). The expansion of the IR region causes borderline genes to enter this region. Because of the reverse repeatability of this region, complete genes or incomplete gene fragments are formed in the IR region on the other side. The comparison of IR boundaries among 14 Sabiaceae chloroplast genomes showed that there was no significant difference in the contraction and expansion of IR regions among 13 *Sabia* chloroplast genomes, with a variation of only 1–6 bp. However, the contraction and the expansion of IR boundaries differed in *M.* aff. *cuneifolia* of the same family and different genera, which also led to the existence of two incomplete pseudogenes (*ycf1* and *rps19*) in the species of genus *Sabia*, but only *ycf1* pseudogene in *M.* aff. *cuneifolia*. Therefore, the variation in the size of chloroplast genomes of the genus *Sabia* is less affected by IR contraction and expansion. Insertion and deletion in IGSs may be the main factors causing variation in size of the *Sabia* chloroplast genome.

Repeat sequences are widespread in chloroplast genomes, and their type, number, and distribution vary according to the species or population. They have been widely used in studies on genetic variation, population structure, and species identification and play an important role in the structural rearrangement of chloroplast genomes (Cavalier-Smith, 2002; Perdereau et al., 2014; Asaf et al., 2016). In this study, SSRs and interspersed repeats in the chloroplast genomes of the genus *Sabia* were preliminarily analyzed to provide a basis for further molecular marker development and intraspecific and interspecific diversity studies of this genus.

### Highly variable regions of *Sabia*

Based on the comparative analyses of mVISTA and nucleotide diversity, 11 highly variable fragments with higher Pi values were extracted for analysis with four universal chloroplast DNA barcodes. The results indicated that none of these universal DNA barcodes conferred a higher discriminatory power than the highly variable fragments screened. Among these regions, *trnH*-*psbA*, with the highest Pi value, is one of the fragments generally recommended as a universal DNA barcode in plants. It has been confirmed to have an effective discrimination rate, and the use of this fragment in combination with other regions can significantly improve the discrimination rate among species (Li et al., 2015; Mishra et al., 2016). In recent years, several studies have explored the applicability of *trnH*-*psbA* fragment in identifying species within the genus *Sabia* (Sui et al., 2011; Yan et al., 2020b), but the results have shown that the discriminatory power of this fragment is not entirely satisfactory. The *trnH*-*psbA* fragment showed a high nucleotide diversity among *Sabia* species, but it was short in length and lacked adequate variation sites, which may explain why this fragment cannot be used alone for the identification of this genus. For loci able to discriminate the genus *Sabia*, it may be necessary to excavate fragments of a certain length and variability at the same time. In the region from *ndhF* to *ndhD* (*ndhF*-*rpl32*-*ccsA*-*ndhD*), the whole fragment showed a continuous high variation, with the exception of the exon regions. Analysis of this fragment also revealed that it was rich in variable sites, parsimony information sites, and InDels. Therefore, the *ndhF*-*ndhD* fragment has the potential to be used as a specific DNA barcode for identifying *Sabia* species or a marker for determining genetic diversity.

For species identification, however, a specific DNA barcode lacks generalizability for use on different fragments for different species. To resolve the problem of the limited resolution of universal DNA barcodes for closely related species, the use of a super-barcode was proposed. This is a method for the rapid and accurate identification of species using complete sequences of chloroplast genomes (Kane and Cronk, 2008; Slipiko et al., 2020). The use of chloroplast genomes has been reported to be effective for discriminating a particular species from a series of genera (Cai et al., 2021; Ji et al., 2021; Wu et al., 2021). In the two duplicate samples that we used, both phylogenetic and genetic distance analyses indicated that these two duplicate samples could be effectively segregated from different species. Nevertheless, in this study, there were too few samples to verify the applicability of the super-barcode in this genus. Thus, there is a need for further research to sequence the chloroplast genome of more species in *Sabia* to build a comprehensive barcode database.

### Phylogenetic relationships and divergence time

To preliminarily explore the relationship within the genus *Sabia*, ML, MP, and BI methods were used for phylogenetic analysis. In the phylogenetic trees, *S. campanulata* subsp. *ritchieae*, *S. yunnanensis*, and *S. schumanniana* belonging to Sect. *Pachydiscus* formed a monophyletic group, which was consistent with the traditional morphological classification. However, this branch was embedded within the species of Sect. *Sabia*, *i*.*e*., the samples of Sect. *Pachydiscus* and Sect. *Sabia* did not form two separate monophyletic groups. Several previous studies have sequenced some gene fragments of *Sabia* for the purpose of species identification or phylogeny reconstruction. In the phylogenetic tree constructed by Yang et al. (2018) based on six chloroplast gene fragments (*atpB*, *rbcL*, *matK*, *ndhF*, *atpB-rbcL*, and *trnL-trnF*), 13 species were divided into two branches. The first branch consisted of *S. swinhoei*, *S. limoniacea*, *S. pauciflora*, and *S. fasciculata*, while the second branch was subdivided into two subclades consisting of *S. paniculata*, *S. philippinensis*, and *S. parviflora* in one subclade and *S. campanulata* subsp. *ritchieae*, *S. dielsii*, *S. transarisanensis*, *S. yunnanensis* subsp. *latifolia*, *S. discolor*, and *S. japonica* var. *sinensis* in another subclade. In the ML phylogenetic tree constructed by Yan et al. (2020a) based on ITS2, *S. schumanniana*, *S. dielsii*, *S. discolor*, *S. campanulata* subsp. *ritchieae*, *S. yunnanensis*, and *S. transarisanensis* have a closer relationship and are clustered with *S. fasciculata*, *S. parviflora*, and *S. swinhoei* in the outer order. Although the species analyzed were not identical across studies and some species had different phylogenetic positions, the results showed that all samples from Sect. *Pachydiscus* in these phylogenetic trees were embedded in the samples from Sect. *Sabia*. These two sections do not form two monophyletic groups. The results on the phylogenetic relationships of the genus *Sabia* obtained in this study might provide new insights for resolving the classification problem of this genus. However, insufficient sampling in this study prevented the provision of sufficient evidence for further revision of *Sabia*. Thus, further research based on expanded sampling is needed to test the phylogenetic relationships and perform taxonomy using more molecular data.

The family Sabiaceae is a group with an amphi-Pacific tropical disjunct distribution. Based on six chloroplast gene fragments, Yang et al. (2018) speculated that this family may had a Eurasian origin in the late Cretaceous and underwent boreotropical range expansion during the Paleogene. With the climatic cooling after the late Miocene, southward migrations from continental Eurasia to Asia and from Central America to South America were inferred. In our study, divergence times were estimated based on RelTime, a program outperforming many other dating methods while using less computational power (Tamura et al., 2012). The results showed that the divergence times of the order Proteales and the family Sabiaceae are similar to those of Yang et al., but our estimation gives a more recent time for the origin of the genus *Sabia*. The *Sabia* samples used in our analysis included only those distributed in China, and chloroplast genome data for other genera of the family Sabiaceae are also insufficient, which may primarily explain the difference in the estimated divergence time. This study involves a preliminary exploration to estimate the species divergence times of the family Sabiaceae based on the chloroplast genome. However, there is a need for further research to collect more complete chloroplast genome sequences of this taxon and combine evidence, such as fossil records, to further understand its evolutionary history.

### Discussion of three samples of *Sabia*

During field survey and observation, some samples with certain peculiarities were found. We sequenced these samples in order to supplement more chloroplast genomic data for *Sabia* species. These samples are briefly discussed below, with the aim of promoting research on *Sabia*.

S. *swinhoei* is a species exhibiting a large variation in the wild. Two samples of *S. swinhoei* were collected from the Medicinal Botanical Garden of GZUTCM, which were introduced a few years ago without reproduction. Observations over recent years have found some variation in the reproductive organs of *S. swinhoei* (JY-2), with the pedicels, calyxes, and petals appearing purplish-red, unlike the usual green color (**Supplementary Figure S4**). In the phylogenetic trees, the two samples of *S. swinhoei* clustered into a monophyletic group with K2p genetic distance of 0. The revision of Flora of China in 2007 supplemented the characteristics of *S. swinhoei* with the presence of purple petals (Guo and Anthony, 2007). In our study, two samples of *S. swinhoei* were introduced into the same botanical garden, but the other plants near them differed, resulting in a great difference in the degree of light that they received. *S. swinhoei* (JY-2) was barely shaded, while *S. swinhoei* (JY-1) received much weaker light. In the wild, *S. swinhoei* inhabits valley forests (Guo and Anthony, 2007). As a woody climber, *S. swinhoei* is inevitably exposed to only limited sunlight in forests with complex environments and a wide range of vegetation types. Therefore, we tentatively speculated that the purplish-red color of pedicels, calyxes, and petals of *S. swinhoei* (JY-2) may be related to the higher exposure to light. *S. swinhoei* (JY-2) may reflect an ecotype produced under special circumstances.

*S.* sp. (CY-1) is a woody climber and deciduous. Its stem is cylindrical, with young branches that are yellowish-green and old branches that are purplish-brown. The leaf blade is nearly papery; ovate-elliptic, apex acuminate, base rounded; adaxially dark green, abaxially light green; glabrous. The cymes are three- to four-flowered. It is similar to *S. dielsii*, but they differ in certain features of the leaves, flowers, and other organs (**Supplementary Figure S5**)—for example, the leaves of *S.* sp. (CY-1) are wider than those of *S. dielsii*. As for the reproductive organs, the petal apex of *S.* sp. (CY-1) is more rounded, with slender bracts, while the petal apex of *S. dielsii* is more acuminate, and the bracts are wider and slightly triangular. Phylogenetic analysis showed that *S.* sp. (CY-1) is a sister to *S. dielsii*, with a genetic distance of 0.0002424, which is shorter than that between the two samples of *S. parviflora* (0.0003645). *S.* sp. (CY-1) was introduced into the Medicinal Botanical Garden of GZUTCM after its discovery. Observations in recent years have shown that its morphology is stable, and it is a population distributed within a certain region. Therefore, there is a need for further research to determine whether this suspected species can be revised into a variety of *S. dielsii*.

*S.* sp. (CY-2) is a woody climber. The stem is cylindrical, the young branches are green, and the old branches are brown, with brown pilose. The leaf blade is nearly leathery; ovate-elliptic, apex acuminate or acute, base rounded; abaxially, adaxially and petiole shortly pilose. Cymes; peduncle with densely yellow-brown pubescent; petals narrowly triangular, yellowish green to white; schizocarp suborbicular, densely pubescent (**Supplementary Figure S6**). The morphological characteristics of *S.* sp. (CY-2) are similar to those of *Sabia ovalifolia* S. Y. Liu found by S. Y. Liu (Liu, 2002) in Guangxi Province, China. Liu pointed out that *S. ovalifolia* is similar to *S. swinhoei*, but *S. swinhoei* is easily distinguished from *S. ovalifolia* by its long, straight pilose branchlets, red spots on the calyx, and undivided ovary. However, this species was not considered to be established in the subsequent revision (Guo and Anthony, 2007). Specifically, *S. ovalifolia* was subsumed as a synonym of *S. swinhoei*. Phylogenetic analysis in this study indicated that *S.* sp. (CY-2) is not sister to *S. swinhoei* but closely related to *S. limoniacea*, which is more dissimilar morphologically. Thus, there is a need for further research to perform detailed anatomical observation and investigation of geographical distribution. Combined with the chloroplast genome data provided in this study, the taxonomic position of this suspicious species will be determined.

## Conclusion

In this study, the chloroplast genomes of 11 *Sabia* samples (including eight species, two suspicious species, and one duplicate sample) were assembled and analyzed. The repeated sequences and highly variable regions of this genus were also analyzed and compared. Fragments with high variation were screened, providing data that can act as a foundation for the analysis of genetic diversity and development of molecular marker for this genus. Through phylogenetic trees, genetic distance, and divergence time, the genetic relationships of the genus *Sabia* were preliminarily explored, providing a basis for comprehensively exploring phylogenetic relationships, solving the classification and identification problems, and exploring the evolutionary history of this genus.

## Data availability statement

The data presented in the study are deposited in the GenBank repository, accession number OP310790–OP310800. Raw data can be found at NCBI under accession number PRJNA899473.

## Author contributions

QS and WX conceived the study. QS and BW collected the samples. QC performed the experiments and data analysis. QC and YH composed the manuscript. YH, CC, and ZW revised the manuscript. All authors contributed to the article and approved the submitted version.
