# Supplementary material for: Complete chloroplast genomes of 11 Sabia samples: Genomic features, comparative analysis, and phylogenetic relationship
Source: Front Plant Sci. 2022 Dec 16;13:1052920. doi: 10.3389/fpls.2022.1052920 (PMC9800934; doi:10.3389/fpls.2022.1052920)
Supplement: Supplementary file 1 [file DataSheet_1.docx]

Supplementary Material

## Supplementary Figures


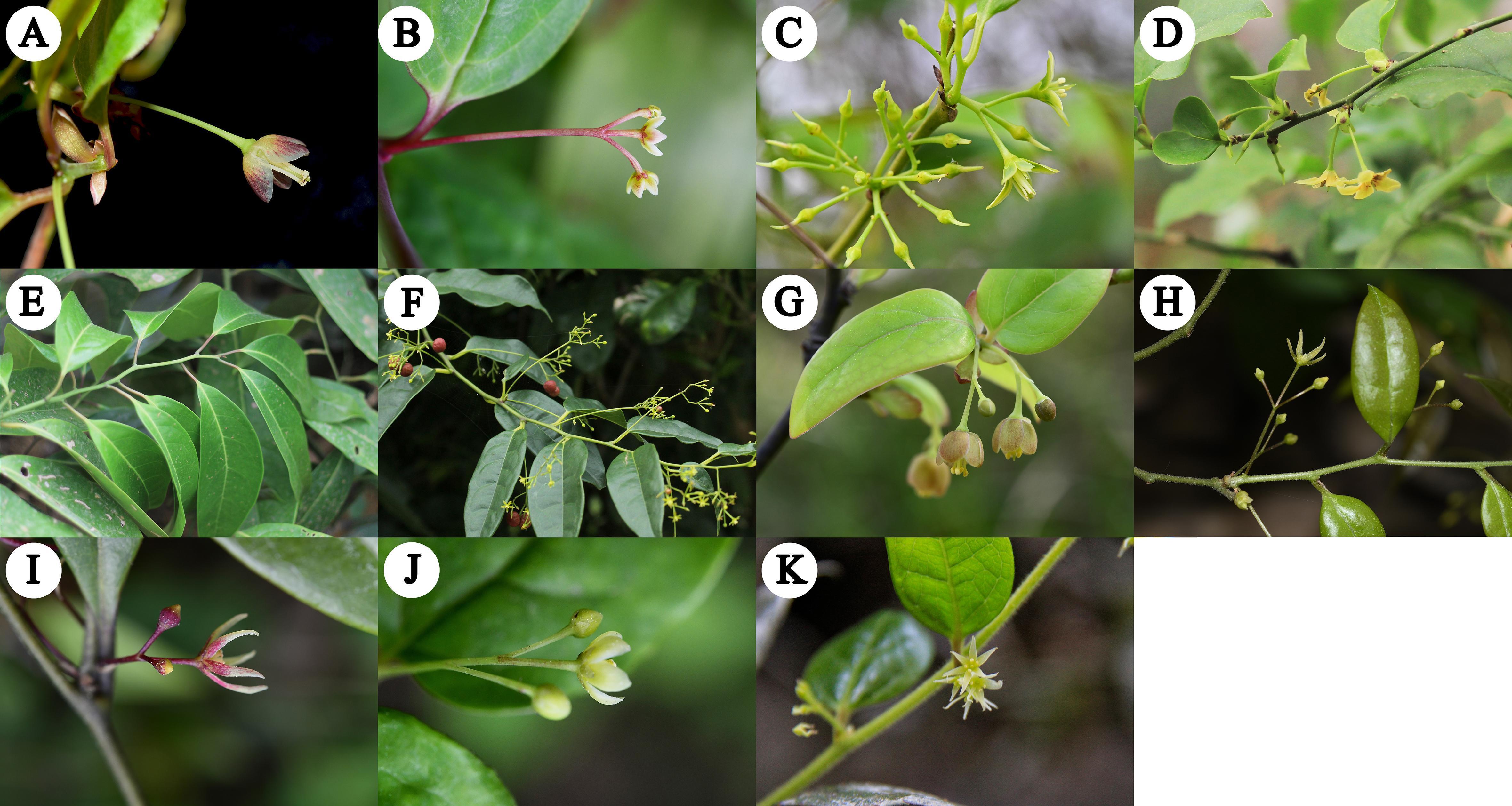


**Supplementary Figure S1. Plant morphology of the *Sabia* species in this study.**

A. *S. campanulata* subsp. *ritchieae*. B. *S. dielsii*. C. *S. fasciculata*. D. *S. japonica*. E. *S. limoniacea*. F.*S. parviflora* (XH-1). G. *S. schumanniana*. H. *S. swinhoei* (JY-1). I. *S. swinhoei* (JY-2). J. *S.* sp. (CY-1). K.*S.* sp. (CY-2).


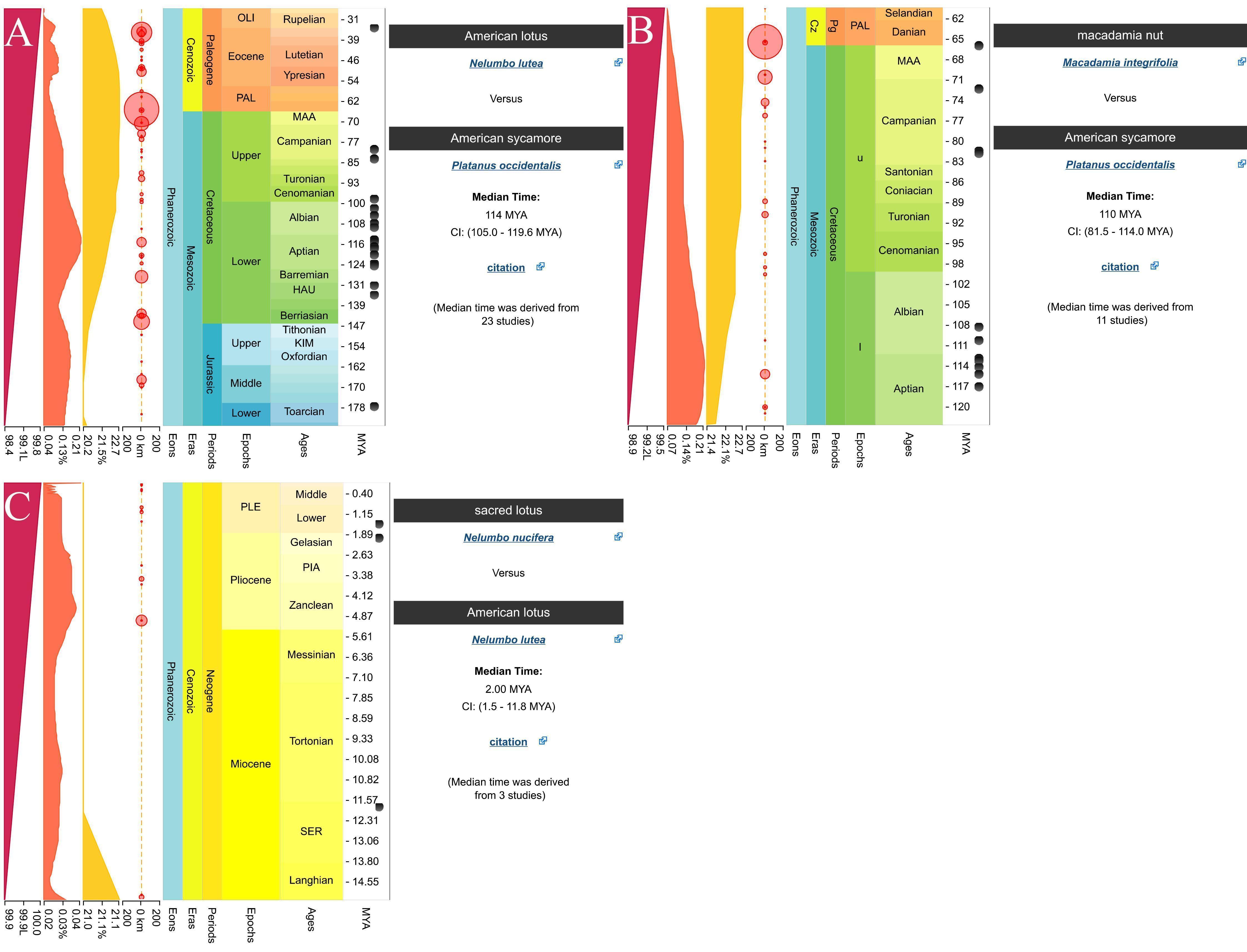


**Supplementary Figure S2. Calibration constraints for divergence times estimation.**

A. *Nelumbo lutea* vs. *Platanus occidentalis*. B. *Macadamia integrifolia* vs. *Platanus occidentalis*. C. *Nelumbo nucifera* vs. *Nelumbo lutea*.

Source: Timetree (www.timetree.org)

**
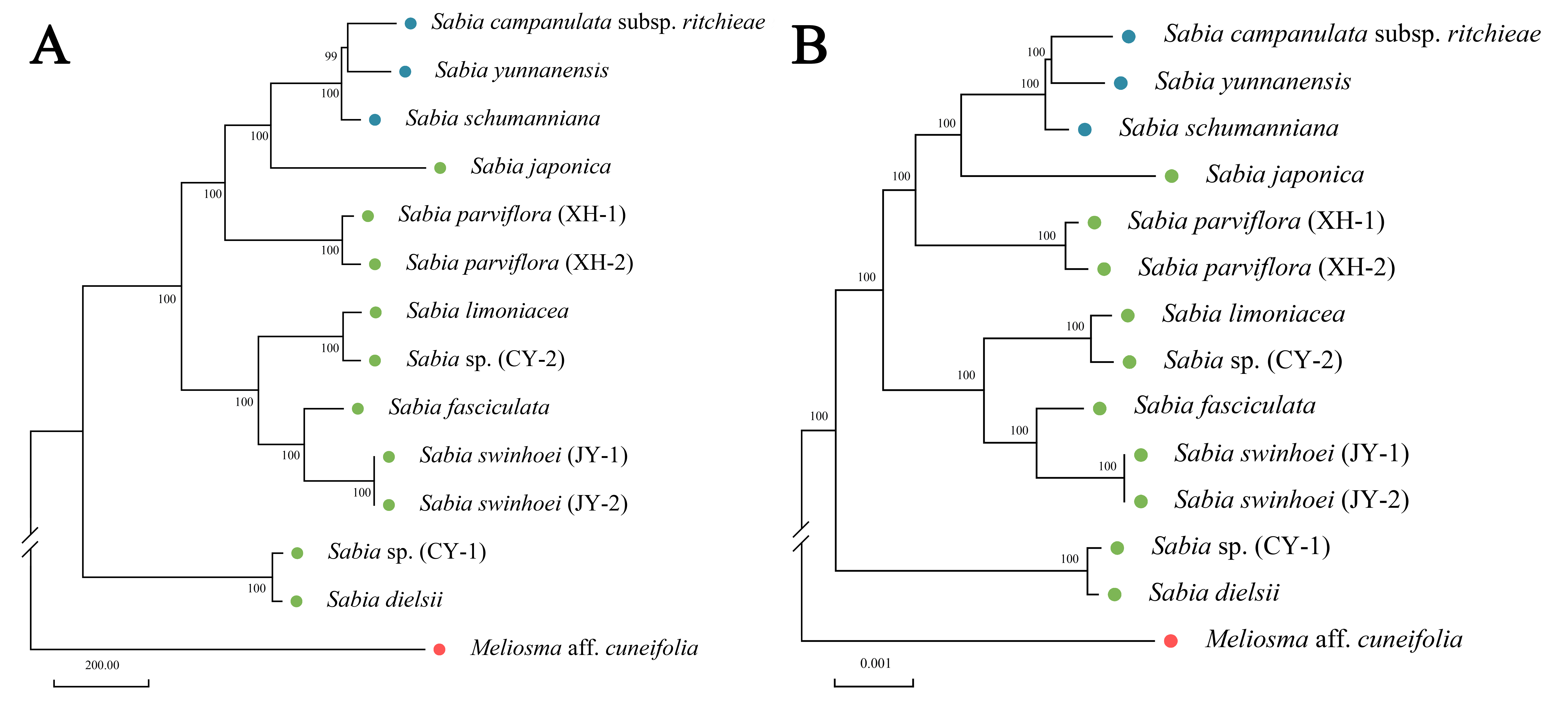
**

**Supplementary Figure S3. Phylogenetic tree obtained using the maximum parsimony (MP) and Bayesian Inference (BI) method for *Sabia* species.**

Numbers above branches indicate MP bootstrap supports (A) and Bayesian posterior probabilities (B).

**
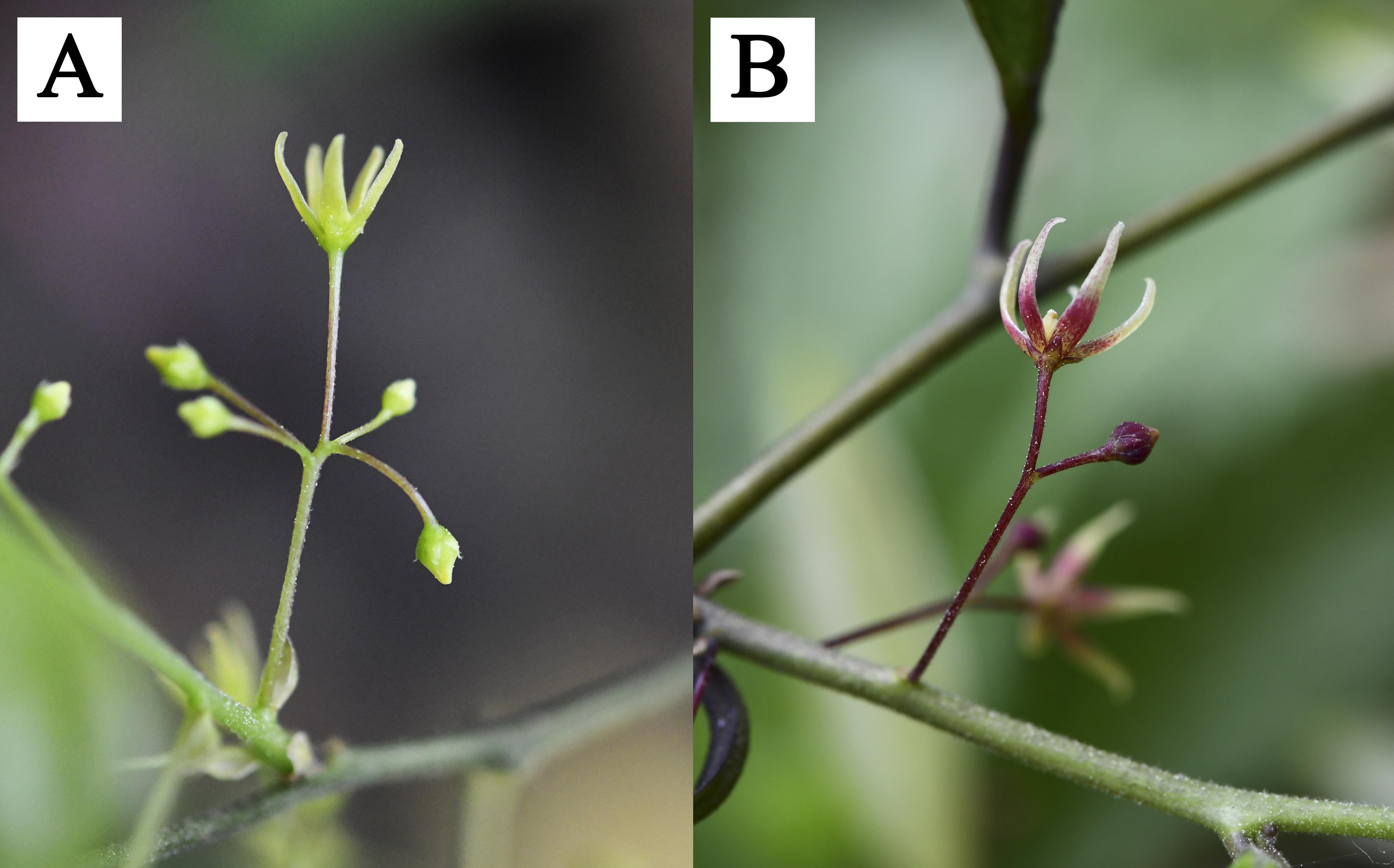
**

**Supplementary Figure S4. Morphology characteristics of *Sabia swinhoei***

A. Inflorescence of *Sabia swinhoei* (JY-1). B.Inflorescence of *Sabia* *swinhoei* (JY-2).

**
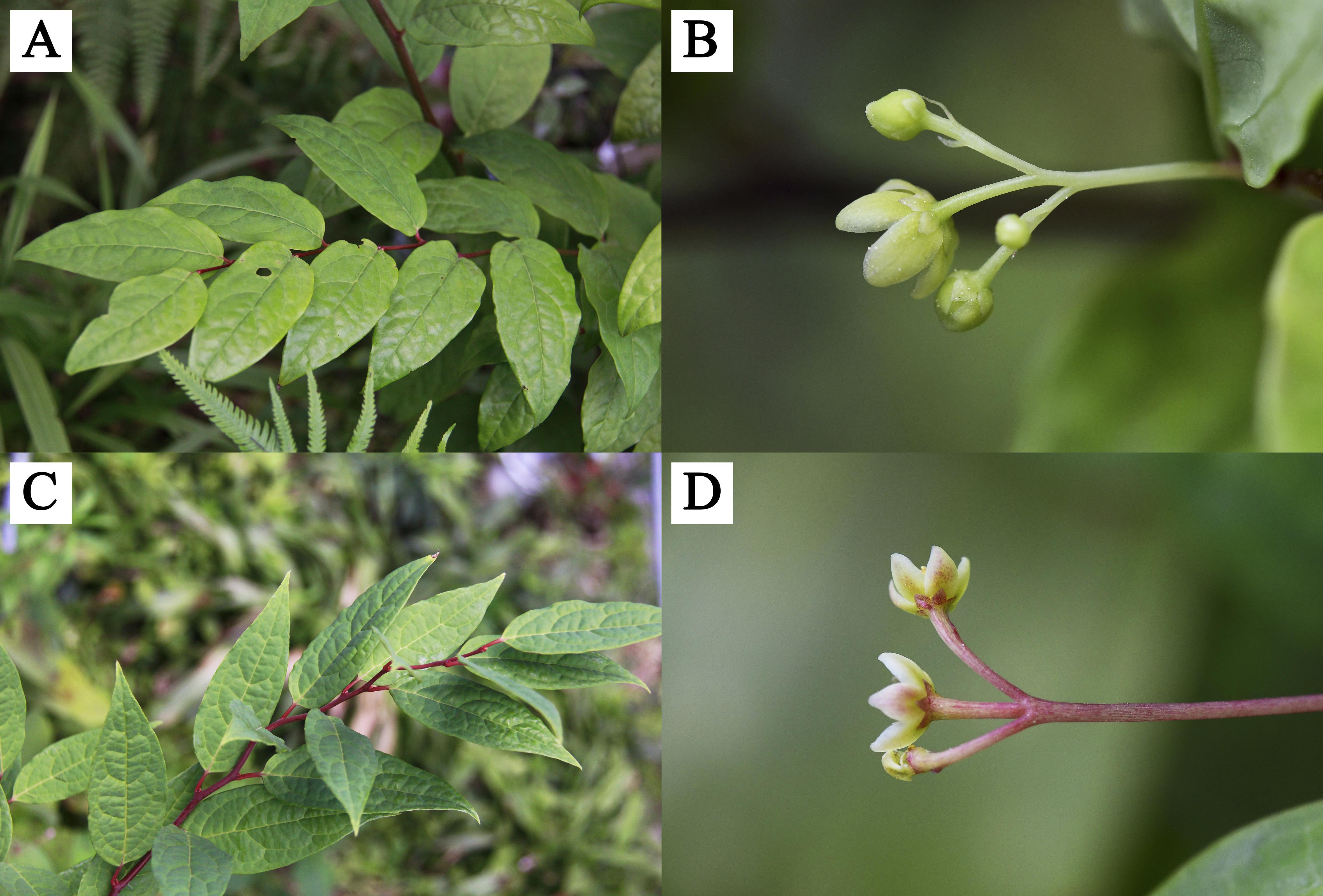
**

**Supplementary Figure S5. Morphology characteristics of *Sabia* sp. (CY-2) and *Sabia dielsii***

A. Branchlet of *Sabia* sp. (CY-1). B. Inflorescence of *Sabia* sp. (CY-1). C. Branchlet of *Sabia dielsii*. D. Inflorescence of *Sabia dielsii*.

**
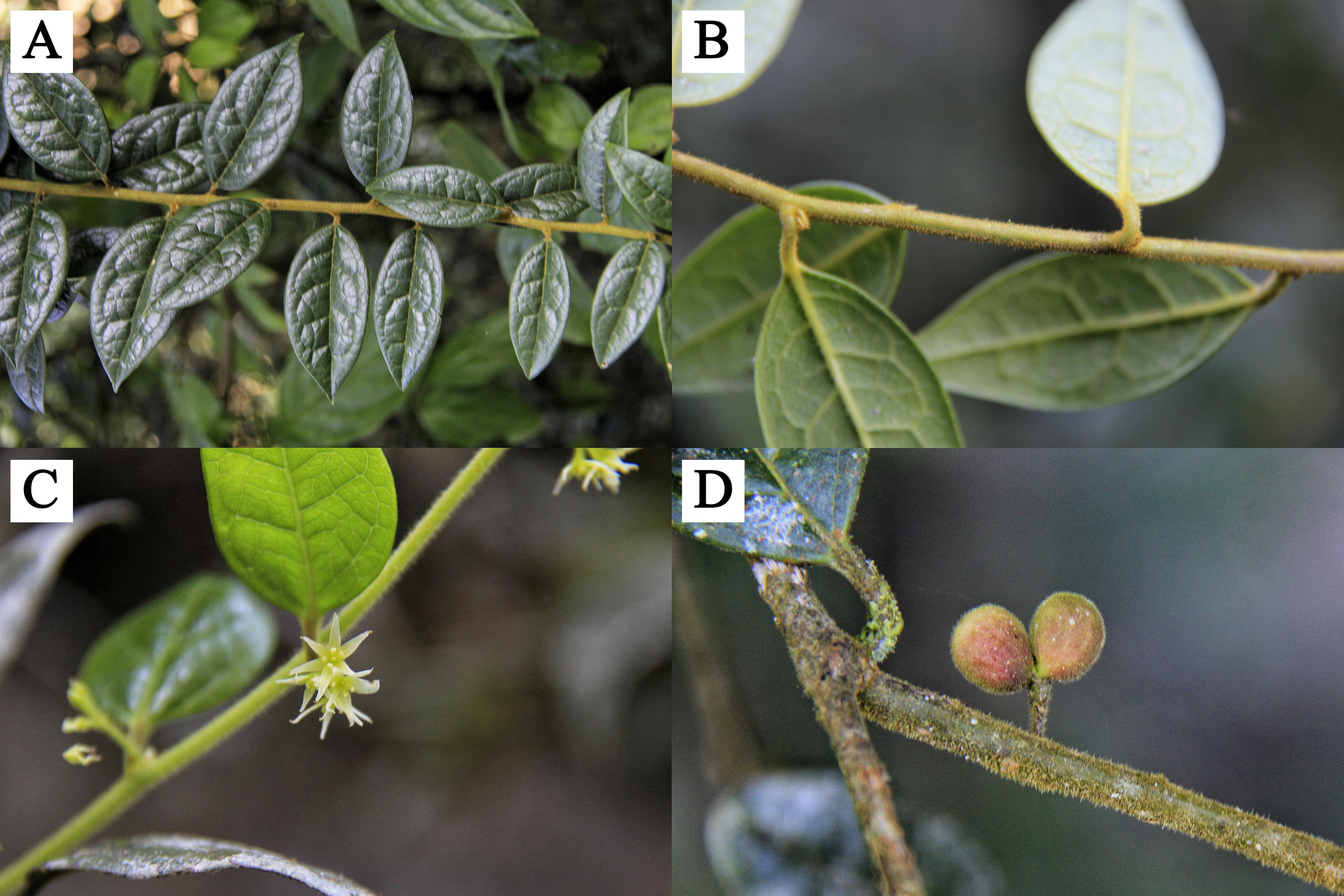
**

**Supplementary Figure S6. Morphology characteristics of *Sabia* sp. (CY-2).**

A. Branchlet of *Sabia* sp. (CY-2). B. Stem of *Sabia* sp. (CY-2) with brown pilose. C. Inflorescence of *Sabia* sp. (CY-2). D. Fruits of *Sabia* sp. (CY-2).

## Supplementary Tables

**Supplementary Table S1. Species information used for divergence time estimation**

| No. | APG IV order | Family | Species | GenBank accession number |
| --- | --- | --- | --- | --- |
| 1 | Proteales | Sabiaceae | *Sabia yunnanensis* Franch. | NC_029431.1 |
| 2 | Proteales | Sabiaceae | *Sabia parviflora* Wall. | NC_059863.1 |
| 3 | Proteales | Sabiaceae | *Meliosma veitchiorum* Hemsl. | MW800950.1 |
| 4 | Proteales | Sabiaceae | *Meliosma cuneifolia* Franch. | MW800998.1 |
| 5 | Proteales | Sabiaceae | *Meliosma rigida* Siebold & Zucc. | MW801256.1 |
| 6 | Proteales | Sabiaceae | *Meliosma thorelii* Lecomte | MW801257.1 |
| 7 | Proteales | Sabiaceae | *Meliosma oldhamii* Miq. ex Maxim. | MW801106.1 |
| 8 | Proteales | Nelumbonaceae | *Nelumbo lutea* Willd. | NC_015605.1 |
| 9 | Proteales | Nelumbonaceae | *Nelumbo nucifera* Gaertn. | NC_025339.1 |
| 10 | Proteales | Platanaceae | *Platanus occidentalis* L. | NC_008335.1 |
| 11 | Proteales | Platanaceae | *Platanus* x *hispanica* Mill. ex Münchh. | MZ128519.1 |
| 12 | Proteales | Proteaceae | *Grevillea robusta* A. Cunn. ex R. Br. | NC_059033.1 |
| 13 | Proteales | Proteaceae | *Macadamia integrifolia* Maiden & Betche | NC_025288.1 |
| 14 | Proteales | Proteaceae | *Heliciopsis lobata* (Merr.) Sleumer | NC_060493.1 |
| 15 | Proteales | Proteaceae | *Orites excelsus* R. Br. | KT716500.1 |
| 16 | Proteales | Proteaceae | *Helicia cochinchinensis* Lour. | MW801291.1 |
| 17 | Proteales | Proteaceae | *Helicia reticulata* W.T. Wang | MW801290.1 |
| 18 | Proteales | Proteaceae | *Helicia nilagirica* Bedd. | NC_057271.1 |
| 19 | Proteales | Proteaceae | *Helicia shweliensis* W.W. Sm. | NC_045942.1 |
| 20 | Ranunculales | Berberidaceae | *Epimedium ecalcaratum* G.Y. Zhong | NC_053530.1 |
| 21 | Ranunculales | Ranunculaceae | *Semiaquilegia guangxiensis* Yan Liu & Y. S. Huang | NC_057495.1 |

**Supplementary Table S2. Codon content of 20 amino acid and stop codons of 11 *Sabia* chloroplast genomes.**

| Amino acid | Codons | Number of codons | | | | | | | | | | |
| --- | --- | --- | --- | --- | --- | --- | --- | --- | --- | --- | --- | --- |
|  |  | A | B | C | D | E | F | G | H | I | J | K |
| Phe | UUU | 880 | 878 | 880 | 881 | 880 | 879 | 880 | 883 | 883 | 878 | 882 |
|  | UUC | 589 | 591 | 591 | 586 | 590 | 593 | 589 | 588 | 588 | 591 | 590 |
| Leu | UUA | 759 | 756 | 757 | 759 | 757 | 758 | 759 | 758 | 758 | 756 | 758 |
|  | UUG | 564 | 567 | 565 | 566 | 566 | 564 | 565 | 566 | 566 | 567 | 564 |
|  | CUU | 572 | 575 | 574 | 573 | 571 | 574 | 574 | 572 | 572 | 575 | 572 |
|  | CUC | 224 | 224 | 225 | 229 | 225 | 223 | 223 | 225 | 225 | 224 | 224 |
|  | CUA | 394 | 393 | 389 | 390 | 389 | 393 | 394 | 391 | 391 | 393 | 389 |
|  | CUG | 196 | 197 | 198 | 193 | 200 | 196 | 196 | 197 | 197 | 197 | 200 |
| Ile | AUU | 1044 | 1042 | 1049 | 1048 | 1046 | 1043 | 1042 | 1047 | 1047 | 1042 | 1046 |
|  | AUC | 480 | 484 | 483 | 476 | 484 | 484 | 481 | 484 | 484 | 484 | 484 |
|  | AUA | 709 | 702 | 699 | 706 | 701 | 706 | 708 | 702 | 702 | 702 | 700 |
| Met | AUG | 634 | 635 | 634 | 636 | 634 | 633 | 635 | 636 | 636 | 635 | 634 |
| Val | GUU | 486 | 483 | 482 | 484 | 482 | 487 | 485 | 487 | 487 | 483 | 483 |
|  | GUC | 194 | 196 | 195 | 195 | 197 | 192 | 194 | 194 | 194 | 196 | 196 |
|  | GUA | 533 | 530 | 535 | 535 | 532 | 535 | 533 | 536 | 536 | 530 | 534 |
|  | GUG | 225 | 227 | 224 | 226 | 224 | 225 | 225 | 225 | 225 | 227 | 222 |
| Ser | UCU | 537 | 534 | 543 | 542 | 543 | 536 | 539 | 539 | 539 | 534 | 543 |
|  | UCC | 367 | 372 | 364 | 364 | 365 | 366 | 366 | 368 | 368 | 372 | 364 |
|  | UCA | 456 | 456 | 453 | 455 | 453 | 456 | 454 | 454 | 454 | 456 | 453 |
|  | UCG | 192 | 186 | 192 | 190 | 191 | 193 | 192 | 191 | 191 | 186 | 191 |
| Pro | CCU | 425 | 427 | 425 | 424 | 425 | 425 | 426 | 426 | 426 | 427 | 425 |
|  | CCC | 240 | 237 | 240 | 239 | 240 | 240 | 239 | 239 | 239 | 237 | 239 |
|  | CCA | 323 | 325 | 324 | 323 | 323 | 324 | 322 | 323 | 323 | 326 | 324 |
|  | CCG | 144 | 147 | 144 | 147 | 146 | 146 | 143 | 144 | 144 | 147 | 146 |
| Thr | ACU | 522 | 522 | 523 | 520 | 523 | 518 | 522 | 523 | 523 | 522 | 524 |
|  | ACC | 276 | 273 | 276 | 276 | 275 | 276 | 277 | 277 | 277 | 273 | 274 |
|  | ACA | 394 | 395 | 395 | 392 | 394 | 390 | 393 | 397 | 397 | 394 | 394 |
|  | ACG | 175 | 171 | 175 | 177 | 174 | 177 | 176 | 173 | 173 | 171 | 174 |
| Ala | GCU | 626 | 628 | 625 | 624 | 625 | 625 | 627 | 625 | 625 | 628 | 625 |
|  | GCC | 224 | 225 | 225 | 222 | 225 | 225 | 224 | 225 | 225 | 225 | 225 |
|  | GCA | 382 | 382 | 380 | 384 | 381 | 385 | 383 | 382 | 382 | 382 | 380 |
|  | GCG | 156 | 160 | 159 | 160 | 159 | 159 | 157 | 158 | 158 | 160 | 160 |
| Tyr | UAU | 767 | 767 | 767 | 765 | 768 | 764 | 767 | 770 | 770 | 767 | 767 |
|  | UAC | 186 | 187 | 187 | 186 | 187 | 188 | 186 | 187 | 187 | 187 | 187 |
| Stop | UAA | 39 | 39 | 39 | 39 | 39 | 40 | 39 | 39 | 39 | 39 | 39 |
|  | UAG | 25 | 25 | 25 | 25 | 25 | 25 | 25 | 25 | 25 | 25 | 25 |
| His | CAU | 516 | 519 | 513 | 519 | 513 | 517 | 516 | 513 | 513 | 519 | 513 |
|  | CAC | 157 | 159 | 159 | 159 | 158 | 157 | 157 | 159 | 159 | 159 | 158 |
| Gln | CAA | 714 | 713 | 707 | 716 | 707 | 712 | 714 | 707 | 707 | 712 | 707 |
|  | CAG | 226 | 224 | 224 | 228 | 225 | 223 | 226 | 225 | 225 | 224 | 225 |
| Asn | AAU | 951 | 943 | 957 | 955 | 955 | 954 | 951 | 955 | 955 | 944 | 955 |
|  | AAC | 289 | 289 | 285 | 290 | 286 | 287 | 289 | 284 | 284 | 288 | 286 |
| Lys | AAA | 974 | 977 | 977 | 978 | 977 | 978 | 974 | 976 | 976 | 977 | 977 |
|  | AAG | 379 | 380 | 384 | 379 | 384 | 378 | 379 | 383 | 383 | 380 | 384 |
| Asp | GAU | 866 | 868 | 865 | 861 | 868 | 873 | 864 | 861 | 861 | 868 | 867 |
|  | GAC | 227 | 222 | 223 | 224 | 222 | 224 | 225 | 225 | 225 | 222 | 224 |
| Glu | GAA | 994 | 994 | 998 | 988 | 998 | 994 | 994 | 997 | 997 | 994 | 998 |
|  | GAG | 373 | 374 | 372 | 380 | 374 | 371 | 374 | 372 | 372 | 374 | 374 |
| Cys | UGU | 225 | 222 | 225 | 227 | 225 | 226 | 226 | 227 | 227 | 222 | 225 |
|  | UGC | 88 | 88 | 88 | 88 | 87 | 86 | 87 | 86 | 86 | 88 | 87 |
| Stop | UGA | 21 | 21 | 21 | 21 | 21 | 20 | 21 | 21 | 21 | 21 | 21 |
| Trp | UGG | 463 | 463 | 464 | 465 | 463 | 463 | 463 | 464 | 464 | 463 | 463 |
| Arg | CGU | 351 | 348 | 351 | 350 | 350 | 353 | 351 | 350 | 350 | 348 | 350 |
|  | CGC | 109 | 112 | 111 | 108 | 111 | 107 | 109 | 111 | 111 | 112 | 111 |
|  | CGA | 346 | 345 | 347 | 346 | 345 | 348 | 346 | 348 | 348 | 346 | 345 |
|  | CGG | 134 | 136 | 131 | 132 | 136 | 136 | 134 | 133 | 133 | 136 | 136 |
| Ser | AGU | 412 | 413 | 410 | 406 | 412 | 409 | 410 | 411 | 411 | 413 | 411 |
|  | AGC | 120 | 120 | 122 | 122 | 120 | 122 | 121 | 125 | 125 | 120 | 121 |
| Arg | AGA | 495 | 493 | 500 | 495 | 499 | 497 | 494 | 497 | 497 | 493 | 498 |
|  | AGG | 189 | 192 | 191 | 189 | 189 | 192 | 191 | 192 | 192 | 192 | 189 |
| Gly | GGU | 589 | 586 | 583 | 586 | 586 | 585 | 587 | 587 | 587 | 586 | 585 |
|  | GGC | 192 | 197 | 195 | 192 | 192 | 193 | 194 | 193 | 193 | 197 | 192 |
|  | GGA | 738 | 734 | 734 | 739 | 734 | 738 | 738 | 736 | 736 | 734 | 737 |
|  | GGG | 324 | 329 | 329 | 323 | 329 | 328 | 326 | 327 | 327 | 329 | 329 |
| Amount | - | 26401 | 26399 | 26407 | 26403 | 26405 | 26414 | 26401 | 26421 | 26421 | 26399 | 26405 |

A. *S. campanulata* subsp. *ritchieae*, B. *S. dielsii*, C. *S. fasciculata*, D. *S. japonica*, E. *S. limoniacea*, F. *S. parviflora* (XH-1),

G. *S. schumanniana*, H. *S. swinhoei* (JY-1), I. *S. swinhoei* (JY-2), J. *S.* sp. (CY-1), K. *S.* sp. (CY-2).
